# Novel Functions of the Phosphatase SHP2 in the DNA Replication and Damage Checkpoints

**DOI:** 10.1371/journal.pone.0049943

**Published:** 2012-11-26

**Authors:** Yiu Huen Tsang, Xianxian Han, Wing Yu Man, Nelson Lee, Randy Y. C. Poon

**Affiliations:** Division of Life Science and Center for Cancer Research, Hong Kong University of Science and Technology, Clear Water Bay, Hong Kong; National Cancer Institute, NIH, United States of America

## Abstract

Replication stress- and DNA damage-induced cell cycle checkpoints are critical for maintaining genome stability. To identify protein phosphatases involved in the activation and maintenance of the checkpoints, we have carried out RNA interference-based screens with a human phosphatome shRNA library. Several phosphatases, including SHP2 (also called PTPN11) were found to be required for cell survival upon hydroxyurea-induced replicative stress in HeLa cells. More detailed studies revealed that SHP2 was also important for the maintenance of the checkpoint after DNA damage induced by cisplatin or ionizing radiation in HeLa cells. Furthermore, SHP2 was activated after replicative stress and DNA damage. Although depletion of SHP2 resulted in a delay in cyclin E accumulation and an extension of G_1_ phase, these cell cycle impairments were not responsible for the increase in apoptosis after DNA damage. Depletion of SHP2 impaired CHK1 activation, checkpoint-mediated cell cycle arrest, and DNA repair. These effects could be rescued with a shRNA-resistant SHP2. These results underscore the importance of protein phosphatases in checkpoint control and revealed a novel link between SHP2 and cell cycle checkpoints.

## Introduction

DNA damage can be introduced by external stresses including radiation, antineoplastic agents, or errors generated from DNA replication and transcription. In mammalian cells, these damages are sensed by PI3K-related kinases including ATM (ataxia telangiectasia, mutated), ATR (ATM- and Rad3-related), and DNA-PK (DNA-dependent protein kinase). Phosphorylation cascades involving downstream kinases CHK1, CHK2, and p38 are then initiated to form part of the DNA damage checkpoint, leading to cell cycle arrest, DNA repair, and apoptosis (reviewed in [1]).

Progress in the past decade has uncovered many of the protein kinases involved in checkpoint activation and signal transduction. Although protein phosphatases are increasingly being recognized for their roles in checkpoint control, there is a significant gap in our understanding of the repertoire of players and their regulation. Several phosphatases are known to act directly on checkpoint components. For example, PPM1D (also called PP2Cδ and WIP1) is involved in dephosphorylating ATM, CHK1, CHK2, and p53, hence is implicated in the silencing of the DNA damage checkpoints (reviewed in [2]). Another phosphatase, PTEN, was found to regulate CHK1 localization through the PI3K (phosphatidylinositol 3′-kinase)–AKT pathway [3], [4]. Phosphorylated histone H2AX (γ-H2AX), which forms foci surrounding double-stranded DNA breaks and helps to recruit other checkpoint components and repair proteins, is believed to be dephosphorylated by PP2A [5]. These and other findings have provided a glimpse of the importance of phosphatases in checkpoint control.

SHP2, also known as PTPN11 (protein tyrosine phosphatase non-receptor type 11), contains two tandem NH_2_-terminal SRC homology 2 (SH2) domains (N-SH2 and C-SH2), a catalytic (PTP) domain, and a COOH-terminal tail with tyrosyl phosphorylation sites and a prolyl-rich motif. It is ubiquitously expressed and plays a role in various cell signaling events for a diversity of functions, including mitogenic activation, metabolic control, transcription regulation, survival, migration, and differentiation (reviewed in [6]). Due to the autoinhibitory binding between its N-SH2 domain and PTP domain, the basal catalytic activity of SHP2 is relatively low. Activation of SHP2 requires conformational changes caused by binding of the SH2 domains to tyrosyl-phosphorylated substrates such as IRS1 and GAB1 [7], [8]. Alternatively, phosphorylation of SHP2 on Tyr542 and Tyr580 upon receptor protein tyrosine kinase activation can promote interaction with N-SH2 and C-SH2 domains respectively, thereby relieving the basal inhibition of the PTP [9].

SHP2 potentiates growth factor/cytokines-stimulated signaling pathways in both catalytic-dependent and -independent manner. In particular, its role as a positive regulator of RAS/ERK is well established (reviewed in [10]). SHP2 is also implicated in other signaling pathways including PI3K–AKT, JNK, and NF-κB pathways (reviewed in [11]). Mutations in SHP2 are a cause of Noonan syndrome and LEOPARD syndrome. These are disorders having overlap phenotypic features with other syndromes caused by germline mutations of components of the RAS–MAPK pathway (characterized by dysmorphic facial features, short stature, hypertelorism, cardiac anomalies, deafness, motor delay, and bleeding diathesis) (reviewed in [12]). Mutations of SHP2 have also been linked to various childhood leukaemia including juvenile myelomonocytic leukaemia (JMML) and acute myelogenous leukaemia (AML), making SHP2 the first proto-oncogene identified in the protein tyrosine phosphatase family (reviewed in [11] [13]). Expression of leukaemia-related SHP2 mutants in murine bone marrow increases cell proliferation and hyperactivates ERK and AKT growth pathways [14], [15].

To identify novel phosphatases that regulate the checkpoints that monitor DNA integrity, we have performed RNAi-mediated screens of the human phosphatome. SHP2 was found to be required for checkpoint activation and cell survival in response to a variety of DNA stresses.

## Materials and Methods

### Materials

All reagents were obtained from Sigma–Aldrich (St. Louis, MO, USA) unless stated otherwise.

### shRNA Library and siRNAs

Short hairpin RNAs (shRNAs) were expressed from the vector pKAR1 [16]. Different shRNA constructs were created by annealing pairs of oligonucleotides into *Bbs* I- and *Xba* I-cut pKAR1 as described [17]. Two shRNA constructs were generated for each target. Stealth siRNA targeting SHP2 (siSHP2(a)), CHK1, and control siRNA were obtained from Invitrogen (Carlsbad, CA, USA). Other siRNAs against SHP2 (siSHP2(b)) (GGUUGCUACGGCUUAUCAUTT) and cyclin E (GGAUGUUGACUGCCUUGAATT) were synthesized by Ribobio (Guangzhou, China). Unless specified, siSHP2(a) was used in the experiments.

### DNA Constructs

Plasmids expressing CHK1 shRNA [18] and histone H2B–GFP [19] were as previously described. The *Eco*R I–*Xba* I fragment from FLAG–cyclin E in pUHD-P1 [20] was ligated into pUHD-P3T/PUR [21] to generate FLAG–cyclin E in pUHD-P3T/PUR. BCL2 cDNA (obtained from Geneservice, IMAGE ID: 4511027) was amplified by PCR with the oligonucleotides 5′-AACCATGGCGCACGCTGGGAGAA-3′ and 5′-TGGAATTCTCACTTGTGGCCCAGATA-3′; the PCR product was digested with *Nco* I–*Eco*R I and ligated into pUHD-P3T/PUR to generate FLAG–BCL2 in pUHD-P3T/PUR. SHP2 cDNA was obtained from Addgene (Cambridge, MA, USA). SHP2 cDNA was amplified by PCR with the oligonucleotides 5′-AGAATTCATGACATCGCGGAGATGG-3′ and 5′-GTCTAGATTCATCTGAAACTTTTCTGC-3′; the PCR product was digested with *Eco*R I-*Xba* I and ligated into pUHD-P3T/PUR to generate FLAG–SHP2 in pUHD-P3T/PUR. Mutations for shSHP2-resistant SHP2 was introduced using Quikchange mutagenesis kit (Stratagene, La Jolla, CA, USA) using the oligonucleotides 5′-TCCCGGGTAATCGTCATGACAACGAA-3′ and 5′-CGTTGTCATGACGATTACCCGGGAGTTTT-3′.

### Cell Culture

The HeLa cell line (cervical carcinoma) used in this study is a clone that expressed the tTA tetracycline repressor chimera [22]. HeLa cells stably expressing histone H2B–GFP were generated as described previously [23]. Hep3B cells (hepatocellular carcinoma) were obtained from American Type Culture Collection (Manassas, VA, USA). Cells were propagated in Dulbecco’s modified Eagle’s medium (DMEM) supplemented with 10% (v/v) calf serum (Invitrogen) (for HeLa) or fetal bovine serum (for Hep3B) and 50 U/ml penicillin–streptomycin (Invitrogen) in a humidified incubator at 37°C in 5% CO_2_. To generate cells stably expressing SHP2 shRNA, cyclin E, or BCL2, HeLa cells were transfected with shSHP2(b) in pKAR1, FLAG–cyclin E in pUHD-P3T/PUR, or FLAG–BCL2 in pUHD-P3T/PUR, respectively, and selected with 1 µg/ml of puromycin. After about two weeks, individual clones were isolated and propagated in the absence of puromycin. Cells were transfected with plasmids and siRNAs (10 nM unless stated otherwise) using a calcium phosphate precipitation method [24] and Lipofectamine™ RNAiMAX (Invitrogen), respectively. Cells were treated with the following reagents at the indicated concentration: Adriamycin (200 ng/ml; Calbiochem, San Diego, CA), aphidicolin (5 µg/ml), blasticidin (5 µg/ml; Invitrogen), camptothecin (0.67 µM; Calbiochem), cisplatin (4 µg/ml; David Bull Laboratories, Perth, Australia), etoposide (10 µg/ml), hydroxyurea (1.5 mM), nocodazole (0.1 µg/ml), PHPS1 (20 µM; Santa Cruz Biotechnology, Santa Cruz, CA, USA), SB203580 (10 µM), thymidine (2 mM), U0126 (10 µM), UCN-01 (100 nM), and pan-caspase inhibitor Z-VAD(Ome)-FMK (10 µM; Enzo Life Sciences, Farmingdale, NY, USA). Double thymidine synchronization was performed as described [25]. For enrichment of prometaphase cells, cells released from double thymidine block were treated with nocodazole for 12 h before the mitotic cells were collected by mechanical shake off.

### Ionizing Radiation

IR was delivered with a caesium^137^ source from a MDS Nordion Gammacell 1000 Elite Irradiator. Unless stated otherwise, cells were irradiated with a dose of 15 Gy.

### Antibodies and Immunological Methods

Monoclonal antibody against β-actin [20], cyclin A2 [26], cyclin B1 [23], and FLAG [27] were obtained from sources as described previously. Rabbit polyclonal antibodies against SHP2 (ab10555) and phosphor-CHK1^Ser317^ (ab2834) were from obtained from Abcam (Cambridge, UK). Mouse monoclonal antibodies against CHK1, cyclin E, rabbit polyclonal antibodies against CDC20 and phosphor-histone H3^Ser10^ were obtained from Santa Cruz Biotechnology. Antibodies against phosphor-SHP2^Tyr542^, CDK1^Tyr15^, ATM^Ser1981^, and CDC25C^Ser216^ were from Cell Signaling Technology (Beverly, MA, USA). Antibodies against phosphor-CHK2^Thr68^ (Calbiochem), phosphor-histone H2AX^Ser139^ (Bethyl Laboratory, Montgomery, TX, USA), CDH1 (Zymed Laboratories, San Francisco, CA, USA), and cleaved PARP (BD Biosciences Pharmingen, Franklin Lakes, NJ, USA) were obtained from the indicated suppliers. Immunoblotting were performed as described [28].

### Flow Cytometry

Flow cytometry analysis after propidium iodide staining was performed as described previously [29].

### Bromodeoxyuridine (BrdU) Incorporation Assays

BrdU incorporation assays were performed as described previously [19] except that the cells were pulsed with BrdU for 1 h only.

### Live Cell Imaging and Immunofluorescence Microscopy

Time-lapse microscopy of living cells and confocal microscopy were performed as previously described [30]. For immunofluorescence microscopy, cells grown on poly-L-lysine-treated cover slips were fixed with cold methanol at −20°C for 10 min. The cells were then washed twice with PBS for 5 min each, blocked and permeabilized with 3% BSA and 0.2% Tween 20 in PBS at 25°C for 30 min, and washed two times with PBS for 5 min each. The cells were incubated with primary antibodies at 25°C for 2 h. After washed four times with PBS, the cells were incubated with Alexa Fluor488 goat anti-rabbit IgG (Invitrogen) for 2 h at 25°C. The cells were washed four times with PBS and stained with Hoechst 33342 (0.5 µg/ml in PBS) for 5 min. After washed three times with PBS, the cover slips were mounted with 2% w/v N-propyl-gallate in glycerol.

### Real-time PCR

HeLa cells transfected with shRNAs were harvested and stored at −80°C. Total RNA was extracted with RNeasy Mini Kit (Qiagen, Hilden, Germany). Reverse transcription was carried out with High-Capacity cDNA Reverse Transcription Kit (Applied Biosystems, Foster City, CA, USA). RT-PCR was performed by using an ABI7500 Fast Real-Time PCR System in the presence of SYBR-green (Applied Biosystems). Primers for RT-PCR were designed with ABI Primer Expressed 3.0: 5′-AGAATGGAGCCATCCCTAAGC-3′ and 5′-TGATGAAGTCCGCGTTGTTC-3′ (CTDSP1), 5′-TCAGCCAGTGTGGAAAACCA-3′ and 5′-GGGCTGTGATGTGCAAGTTG-3′ (DUSP5), 5′-AATCTACATCAGATCCAGGGTCACT-3′ and 5′-TCTGACTGATTGTTGCAAACTTTG-3′ (DUSP11), 5′-CGGTTGCCCTGGATCTCA-3′ and 5′-CAGAATCCCAAGCATGGACAA-3′ (PPFIA4), 5′-CAGAGAGTGGCAAGCGAAAAC-3′ and 5′-AGGTCCGGAGGGCTCAGA-3′ (PPM1G), 5′-GAATATGGCGTCATGCGTGTT-3′ and 5′-CCGTATTCCCTTGTCCAACCT-3′ (SHP2), 5′-GCCTCAGCAGATAGCTTCATAAAAT-3′ and 5′-CCCTGTCCGGCCTATGC-3′ (PTPN20A), 5′-GGGAAATCGTGCGTGACATT-3′ and 5′-GGAACCGCTCATTGCCAAT-3′ (actin). The mRNA expression was normalized to actin.

## Results

### Whole Phosphatome Screens Reveal Downregulation of Several Phosphatases Increases Sensitivity to Hydroxyurea

We generated a library of short hairpin RNAs (shRNAs) targeting human phosphatases. All catalytic subunits of the phosphatome and some of the regulatory subunits and interacting proteins were represented (215 genes in total). For each gene in the library, two shRNAs that targeted different regions of the mRNA were designed (Table S1).

To identify phosphatases involved in checkpoints that monitor DNA stress, HeLa cells were transfected with a mixture of the two shRNAs against individual phosphatases before challenged with hydroxyurea (HU) for 48 h. A plasmid expressing histone H2B–GFP was cotransfected for monitoring the morphology of the transfected cells (Fig. S1A). Cells with defective checkpoints were expected to undergo unscheduled cell cycle progression in the presence of HU, resulting in an increase of cell death.

To verify that the assay could in principle identify checkpoint regulators, we first used shRNAs against CHK1 (shCHK1) to disrupt the checkpoint (Fig. S1B and S1C). As expected, HU induced a cell cycle arrest in vector-transfected cells. In contrast, massive cell death was found in shCHK1-expressing cells. Consistent with the disruption of the checkpoint, histone H3^Ser10^ phosphorylation (an indication of mitosis) was increased in shCHK1-transfected cells (Fig. S1D, lane 8).

**Figure 1:**
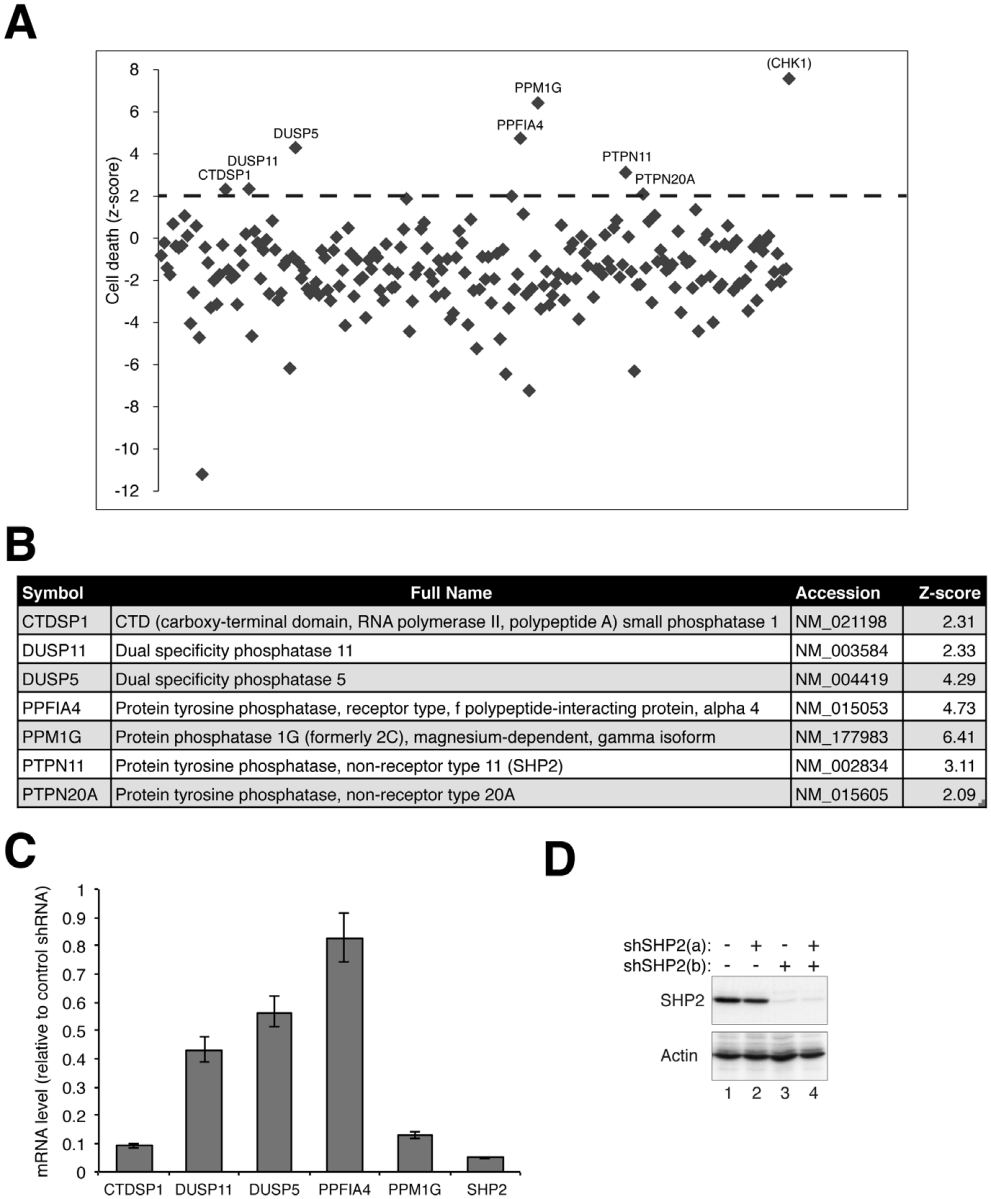
Identification of phosphatases required for cell survival during the activation of the DNA replication checkpoint. (**A**) Screening of phosphatases required for maintain the DNA replication checkpoint. HeLa cells were transfected with a library of plasmids expressing shRNAs against the human phosphatome. A plasmid expressing histone H2B–GFP was co-transfected. At 48 h after transfection, the cells were incubated in the presence of HU for another 48 h. Cell death in the transfected cells was quantified under fluorescence microscopy. The mean was calculated from vector control. The names of the clones with z-score>2 are indicated. CHK1 shRNA was used as a positive control. (**B**) Summary of clones with z-score>2. (**C**) The decrease of mRNA level by shRNA. The efficacy of the shRNA-mediated knockdown of mRNA was determined with RT-PCR. HeLa cells were transfected with plasmids expressing shRNAs against the indicated phosphatases. A plasmid expressing a blasticidin-resistant gene was co-transfected. Transfected cells were enriched by blasticidin treatment for 36 h. Total RNA was prepared and the levels of mRNA were analyzed with RT-PCR (mean±SD of three independent experiments). (**D**) Downregulation of SHP2 protein with shRNA. Cells were transfected with the two SHP2 shRNAs either individually or in a mixture and treated as in panel C. Lysates were prepared and the expression of SHP2 was detected with immunoblotting. Uniform loading of lysates was confirmed by immunoblotting for actin.

Subsequent screening with the library indicated that depletion of several phosphatases resulted in an increase of cell death after HU treatment (Fig. 1A).

To avoid false-positives originated from phosphatases that are essential for normal growth, the percentage cell death after HU treatment was subtracted from that without HU challenge as the basis for calculating the z-score. Seven candidates increased cell death over a z-score of 2 (Fig. 1B). To verify that the mRNAs of these candidates were reduced by the shRNAs, the mRNA levels were analyzed with quantitative RT-PCR (Fig. 1C). Among the seven candidates, PTPN20A mRNA was not detected by RT-PCR, probably due to a very low expression in HeLa cells. Moreover, PPFIA4 (a PTPRF-interacting protein) was not downregulated by the shRNA, suggesting that the effect on cell death was probably non-specific.

One of the candidates, the protein tyrosine phosphatase SHP2 (PTPN11), is one of the first proto-oncogenes identified in the protein tyrosine phosphatase family. Mutations of SHP2 are linked to leukaemia as well as to the relatively common autosomal dominant congenital disorder Noonan syndrome. In this study, we further explored the defects of SHP2-depleted cells.

RT-PCR verified that SHP2 mRNA was downregulated by the shRNAs (Fig. 1C). Downregulation of SHP2 at the protein level was confirmed with immunoblotting (Fig. 1D). Interestingly, only one of the shRNAs was effective in depleting SHP2. Downregulation of SHP2 was equally effective when the two shRNAs were combined (as in the original screening).

Taken together, these screens indicate that several phosphatases, among them SHP2, may be important for the activation or maintenance of the DNA replication checkpoint.

### SHP2 is Important for Cell Survival after Hydroxyurea Challenge

**Figure 2:**
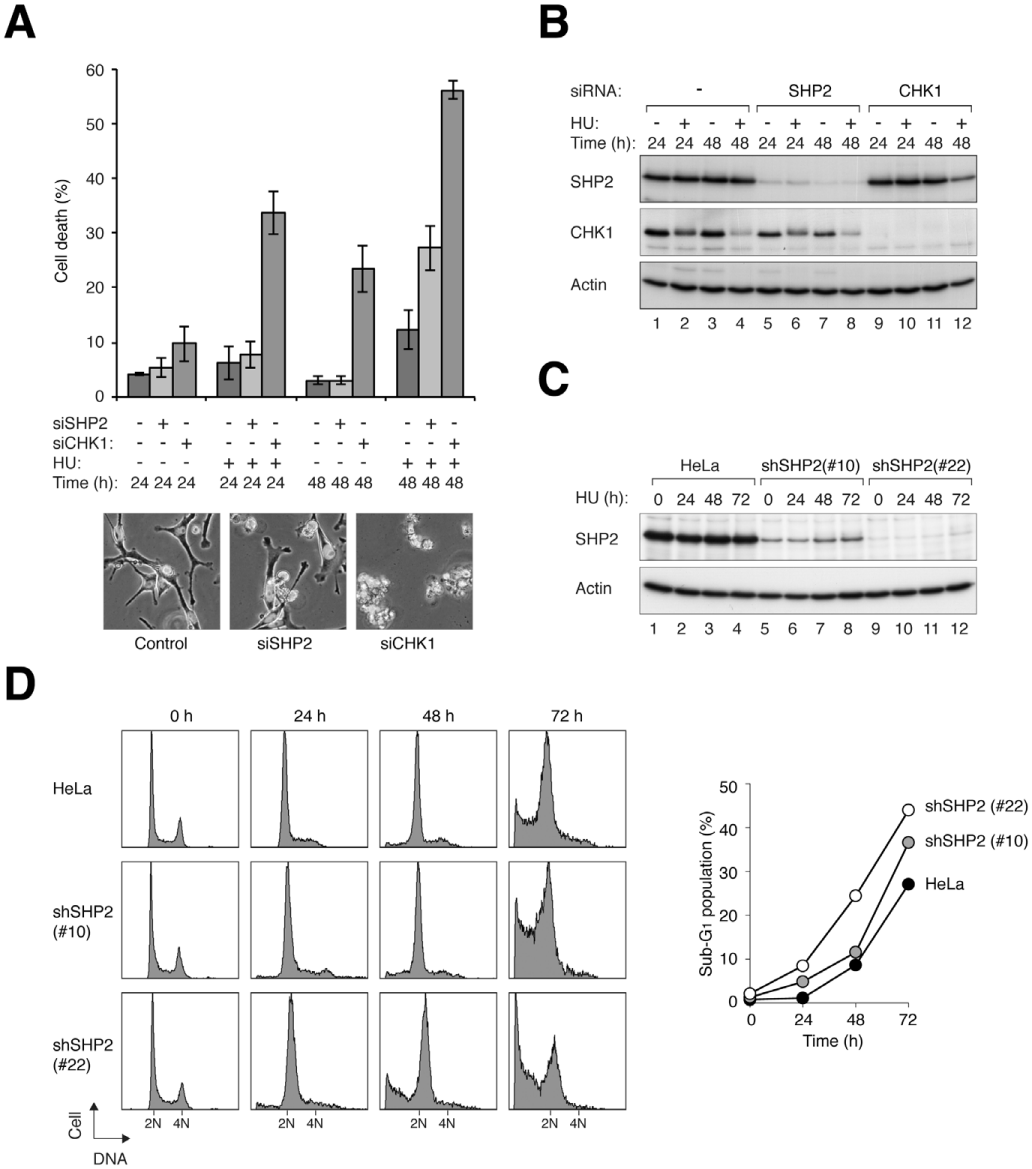
Depletion of SHP2 increases the sensitivity to HU. (**A**) Transfection of SHP2 siRNA increases HU-mediated cell death. HeLa cells were transfected with control, siSHP2, or siCHK1. The cells were treated with HU and the levels of cell death were quantified under fluorescence microscopy (*n* = 200). Mean±SD of three independent experiments is shown. Representative images are shown in the lower panels. (**B**) Validation of SHP2 knockdown. Lysates were prepared from cells treated as described in panel A. The siRNA-mediated knockdown of SHP2 and CHK1 was confirmed by immunoblotting. Note that the signals of CHK1 appear to be weaker after HU treatment because of the multiple phosphorylation-dependent gel mobility shifts. (**C**) Downregulation of SHP2 in cells stably expressing SHP2 shRNA. HeLa cells that stably expressed shSHP2 were generated as described in Materials and Methods. Two shSHP2-expressing clones and the parental HeLa cells were treated with HU and harvested at different time points. Lysates were prepared and the expression of SHP2 was analyzed with immunoblotting. (**D**) Cells stably expressing shSHP2 are more susceptible to HU-induced cell death. Cells were treated as in panel C. At the indicated time points, the cells were harvested and processed for flow cytometry analysis. The levels of sub-G_1_ population were quantified.

To ensure that the effects of SHP2 shRNAs on the HU-induced checkpoint was specific, we next used a chemically-synthesized siRNA as an alternative way to deplete SHP2. The cells were challenged with HU after SHP2 depletion (Fig. 2A). Treatment of HU alone induced some cell death after 48 h (12±2%). As a control, knockdown of the classic checkpoint component CHK1 triggered massive cell death (56±2%). Significantly, depletion of SHP2 with siRNA also enhanced HU-mediated cell death (27±4%), albeit with as lesser severity than the knockdown of CHK1. The depletion of SHP2 by the siRNA was confirmed by immunoblotting (Fig. 2B). Collectively, these data indicated that knockdown of SHP2 with either shRNA or siRNA enhanced HU-mediated cell death.

To exclude the possibility that the increase in HU-mediated cell death was contributed by stress caused by transient transfection, we isolated cell lines that stably expressed shSHP2. Two clones that contained different residual amount of SHP2 were treated with HU and analyzed at different time points (Fig. 2C). Compare to the parental HeLa cells, shSHP2-expressing cells underwent more apoptosis after HU treatment (Fig. 2D). The extent of sub-G_1_ population appeared to correlate with the degree of SHP2 depletion (Fig. 2E).

Taken together, our data suggested that in addition to its well-established role in growth factors-mediated cell proliferation, SHP2 may also play a role in cell survival under HU-induced replication stress.

### Phosphorylation of SHP2^Tyr542^ is Induced after Replication Stress

**Figure 3:**
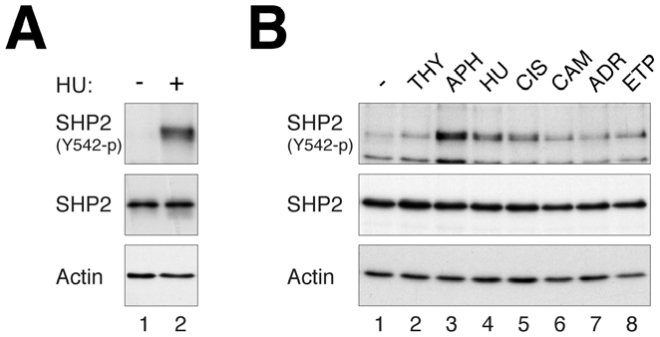
SHP2 is activated by multiple agents that induce DNA replicative stress. (**A**) SHP2^Tyr542^ is phosphorylated after replication stress. HeLa cells were incubated with HU for 64 h. The cells were harvested and immediately boiled in SDS sample buffer to preserve SHP2 phosphorylation. Phosphorylated SHP2^Tyr542^ and total SHP2 were detected with immunoblotting. Uniform loading of lysates was assessed by actin analysis. (**B**) SHP2 is activated by a variety of stresses. HeLa cells were treated thymidine (THY), aphidicolin (APH), HU, CIS (2 µg/ml), camptothecin (CAM), Adriamycin (ADR), or etoposide (ETP). The cells were harvested after 48 h. Phosphorylated SHP2^Tyr542^ and total SHP2 were detected with immunoblotting. Uniform loading of lysates was assessed by actin analysis.

Activation of SHP2 involves the phosphorylation of SHP2^Tyr542^ [9]. We hypothesized SHP2 could also be phosphorylated in response to replicative stress. Using a phosphor-SHP2^Tyr542^-specific antibody, we found that SHP2^Tyr542^ phosphorylation was increased following HU challenge (Fig. 3A).

We next examined if other stress signals that stimulated the DNA integrity could also activate SHP2 (Fig. 3B). Similar to HU, other replication stalling agents including aphidicolin and cisplatin (CIS) were able to induce SHP2^Tyr542^ phosphorylation. In agreement with our data, CIS-induced SHP2 activation has also been demonstrated recently using a chemical probe-based assay [31]. Thymidine was less effective in activating SHP2, probably because thymidine was only effective as a S phase blocker during the first 24 h. DNA damaging agents that mainly induced G_2_ arrest, including camptothecin (topoisomerase I inhibitor), Adriamycin and etoposide (topoisomerase II inhibitors) were less effective in inducing SHP2^Tyr542^ phosphorylation. Collectively, these results indicated that SHP2 could be activated by multiple DNA stress-inducing agents.

### SHP2 is Required for Maintaining Cell Survival after Cisplatin-mediated DNA Damage

**Figure 4:**
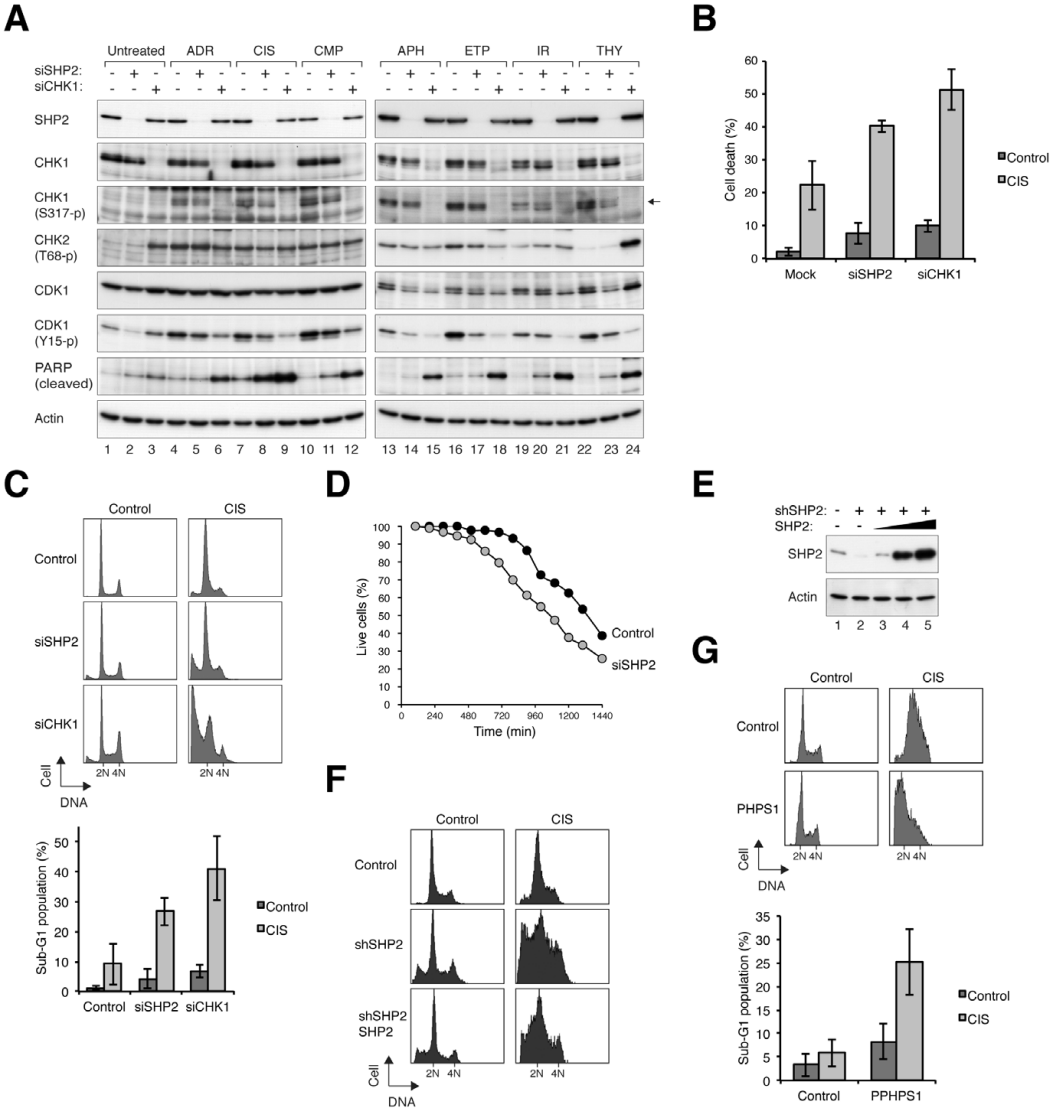
SHP2 is required for cell survival during CIS-activated checkpoint. (**A**) Downregulation of SHP2 increases apoptosis in response to CIS. HeLa cells transfected with control, SHP2, or CHK1 siRNAs were treated with either buffer or CIS for 24 h. Lysates were prepared and analyzed with immunoblotting. The signals of cleaved PARP indicate that SHP2-depleted cells underwent apoptosis after CIS treatment. (**B**) Downregulation of SHP2 enhances CIS-mediated cell death. HeLa cells transfected with control, SHP2, or CHK1 siRNAs were treated with either buffer or CIS. After 24 h, cell death was analyzed with fluorescence microscopy (*n* = 450). Representative photos can be found in Fig. S2A. Mean±SD of three independent experiments is shown. (**C**) Downregulation of SHP2 increases the sub-G_1_ population after CIS treatment. HeLa cells transfected with control, SHP2, or CHK1 siRNAs were treated with either buffer or CIS. After 24 h, the cells were harvested and analyzed with flow cytometry. Quantification of the sub-G_1_ population indicated the siSHP2- and siCHK1-transfected cells were hypersensitive to CIS (bottom panel; mean±SD of three independent experiments). (**D**) Time-lapse imaging of SHP2-depleted cells. HeLa cells stably expressing histone H2B-GFP were transfected with either control or siSHP2. After treatment with CIS, the fate of individual cells were tracked with time-lapse microscopy at 5 min/frame for 24 h (*n* = 100). Analysis of the percentage of surviving cells indicates that SHP2-depleted cells were more sensitive to CIS than control cells. The data of the individual cells can be found in Fig. S2B. (**E**) Expression of a shRNA-resistant SHP2. HeLa cells were transfected with either control vector or shSHP2. Control vector or different amount of SHP2-expressing construct (2.5 µg, lane 3; 5 µg, lane 4; 7.5 µg, lane 5 per 10-cm plate) was co-transfected. After 38 h, the cells were harvested for immunoblotting analysis. (**F**) Expression of SHP2 rescues shSHP2-mediated sensitization of CIS treatment. Cells were transfected with control vector, shSHP2, or shSHP2 together with 2.5 µg of SHP2-expressing construct as in panel (E). The cells were treated with CIS at 38 h after transfection. After another 24 h, the cells were harvested and analyzed with flow cytometry. (**G**) Inhibition of SHP2 with PHPS1 enhances CIS-mediated apoptosis. HeLa cells were treated with either buffer or 20 µM of PHPS1 for 24 h before they were treated with buffer or CIS. After 24 h, the cells were harvested and analyzed with flow cytometry. The sub-G_1_ population was quantified (mean±SD of three independent experiments, bottom panel).

We next investigated if SHP2 is also involved in cell survival in response to different DNA stress-inducing agents. Cells were depleted of SHP2 with siRNA before treated with various agents that triggered DNA damage or replication block (Fig. 4A). As a control, depletion of CHK1 increased apoptosis in response to all the agents (as measured by PARP cleavage). In contrast, depletion of SHP2 selectively increased PARP cleavage in response to CIS treatment. The levels of cleaved PARP was higher after treatment with both siSHP2 and CIS than with either of the treatments alone.

The increase in CIS-induced apoptosis after SHP2 depletion was confirmed using other assays including direct analysis of apoptosis using microscopy (Fig. 4B, representative photos are shown in Fig. S2A). Flow cytometry analysis further verified that CIS treatment significantly increased the sub-G_1_ population in SHP2-depleted cells (Fig. 4C). Finally, we also used time-lapse microscopy to track the fate of individual cells after CIS treatment (Fig. 4D). These analyses confirmed that in response to CIS, SHP2-depleted cells died faster in comparison to control cells. Although the exact percentage of cell death differed with different assays, they were in agreement that siSHP2 significantly increased the cell death induced by CIS. A similar increase in apoptotic cells after SHP2 depletion was observed with Hep3B cells (Fig. S3A), indicating that the effects of siSHP2 on CIS sensitivity was not restricted to HeLa cells.

The specificity of SHP2 depletion on CIS responses were further evaluated in cells that stably expressed shSHP2. Compare to the parental HeLa cells, different shSHP2-expressing clones displayed higher levels of sub-G_1_ cells in response to CIS (Fig. S3B), indicating that the effects on CIS-mediated cell death was not limited to the siSHP2.

A rescue experiment was used to verify the specificity of the effects of the shSHP2. Silence mutations were introduced into a SHP2 construct, rendering it resistant to shSHP2 (Fig. 4E). The cell death caused by shSHP2 and CIS was reduced when the SHP2 construct was co-expressed with the shRNA to a similar level as the endogenous SHP2 (Fig. 4F).

We further used a specific SHP2 inhibitor (PHPS1) [32] to investigate the role of SHP2. The presence of PHPS1 increased the levels of sub-G_1_ cells in response to CIS (Fig. 4G). These data indicate that CIS-mediated cell death was also enhanced when the activity of SHP2 was inhibited without using siRNA or shRNA.

To determine if the CIS-induced cell death was through apoptosis, a pan-caspase inhibitor was added together with CIS. Both the sub-G_1_ population (Fig. S4A) and cleaved PARP (Fig. S4B) induced by siSHP2 and CIS treatments were abolished. Interestingly, even when nocodazole was included to trap SHP2-depleted cells in mitosis, there was only a marginal enrichment of G_2_/M cells (Fig. S4A) and histone H3^Ser10^ phosphorylation (Fig. S4B), indicating that the SHP2-depleted and CIS-treated cells were unable to progress through the cell cycle.

We also generated a cell line that overexpressed anti-apoptotic protein BCL2. Compare to the parental HeLa cells, BCL2-expressing cells were protected from cell death caused with SHP2 depletion. Both the sub-G_1_ population (Fig. S4C) and PARP cleavage (Fig. S4D) induced by siSHP2 and CIS were abolished in the BCL2-expressing cells, again indicating that SHP2 depletion predisposed cells to apoptosis.

Taken together, these results indicated that similar to after HU treatment, depletion of SHP2 promoted apoptotic cell death in response to the DNA crosslinking CIS.

### SHP2 Depletion Delays Cyclin E accumulation and S Phase Entry

**Figure 5:**
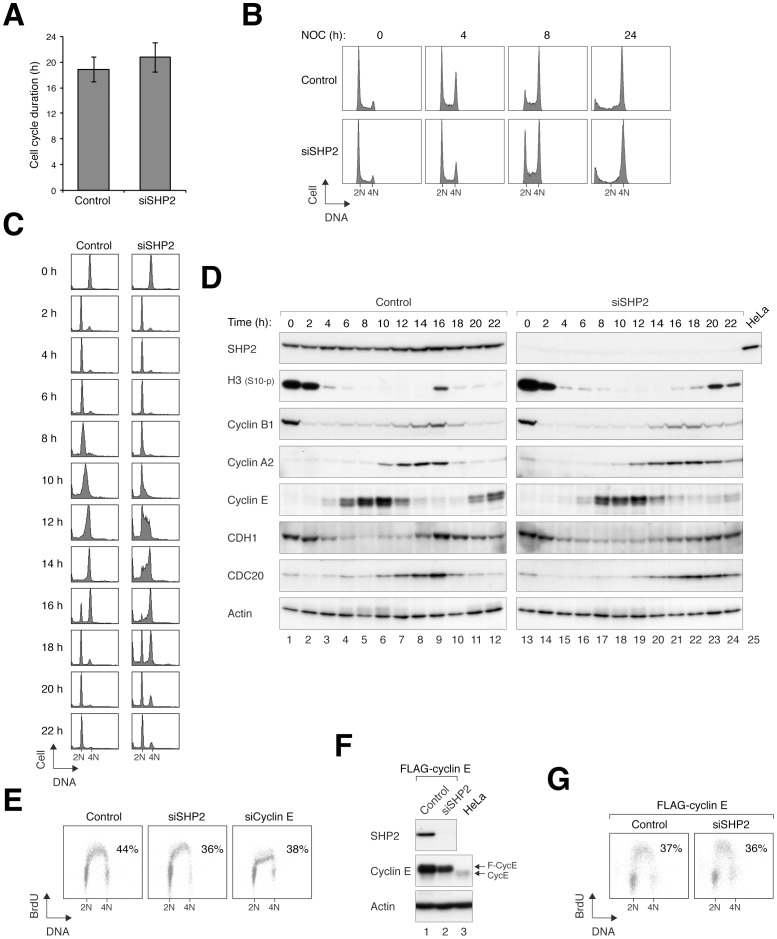
Depletion of SHP2 delays cyclin E accumulation and lengthens the cell cycle. (**A**) Depletion of SHP2 in HeLa cells lengthens the cell cycle by ∼2 h. HeLa cells expressing histone H2B-GFP were transfected with either control (*n* = 37) or siSHP2 (*n* = 23). Individual cells were tracked by time-lapse microscopy for 30 h. The duration of the interphase (from one mitosis to the next) was quantified (mean±SD). The data of the individual cells can be found in Fig. S5A. (**B**) Delay of cell cycle progression after SHP2 depletion. Mock- or siSHP2-transfected HeLa cells were treated with nocodazole and harvested at different time points for flow cytometry analysis. The relatively slow accumulation of G_2_/M population in siSHP2-transfected cells indicates an interphase delay. (**C**) Depletion of SHP2 delays S phase entry. Mock- or siSHP2-transfected HeLa cells were synchronized at mitosis with nocodazole and mechanical shake off. The cells were released into the cell cycle by replating in medium without nocodazole. At the indicated time points, the cells were harvested and analyzed with flow cytometry. SHP2-depleted cells entered S-phase (t = 10) 2 h later than the mock-transfected cells. (**D**) Cyclin E accumulation was delayed in SHP2-deplated cells. Mock- or siSHP2-transfected cells were synchronously released from mitosis as in panel C. The expression of the indicated proteins was detected with immunoblotting. (**E**) Depletion of both SHP2 and cyclin E reduces the S phase population. HeLa cells were transfected with control, SHP2, or cyclin E siRNA. The cells were pulsed with BrdU for 1 h before analyzed with flow cytometry. The percentages of BrdU-positive cells are shown. (**F**) Depletion of SHP2 in cyclin E-overexpressed cells. A HeLa cell line that overexpressed FLAG–cyclin E was generated. The cells were transfected with either control or siSHP2. Lysates were prepared and the expression of cyclin E and SHP2 was detected with immunoblotting. Lysates of the parental HeLa cells were loaded to indicate the level of endogenous cyclin E. (**G**) The effects of SHP2 depletion on S phase is abolished when cyclin E is overexpressed. The FLAG–cyclin E-overexpressed cells were transfected with either control or siSHP2, pulsed with BrdU for 1 h, before analyzed with flow cytometry. The percentages of BrdU-positive cells are shown.

We noted that siSHP2-expressing cells exhibited a slower growth rate than control cells (data not shown). To further characterize this effect, we carried out time-lapse experiments to quantify the duration of the cell cycle of individual cells. The average cell cycle of HeLa cells was extended by ∼2 h after SHP2 depletion (Fig. 5A). Consistent with this, SHP2-depleted cells accumulated in mitosis slower than control cells after addition of nocodazole (Fig. 5B).

To address if cyclin accumulation was involved in the cell cycle delay associated with SHP2 depletion, cells were synchronized at mitosis and released into the cell cycle. Flow cytometry revealed that while the cells progressed from mitosis into G_1_ normally, entry into S phase was delay by more than 2 h (Fig. 5C). Immunoblotting analysis indicated that cyclin E expression during G_1_ was delayed in siSHP2-transfected cells (Fig. 5D). This alteration of cyclin E preceded other cell cycle regulators such as cyclin B1 and cyclin A2 later in the cell cycle. A similar defect of cyclin E accumulation was also observed when SHP2 was depleted with shRNA in the stable cell lines (e.g. see Fig. S3B).

Using BrdU incorporation assay, we found that the fraction of S phase cells was reduced after SHP2 depletion (Fig. 5E). As expected, BrdU incorporation was similarly reduced after depletion of cyclin E (Fig. 5E). Furthermore, cyclin E depletion delayed cell cycle progression similarly as SHP2 depletion (Fig. S5B), suggesting that the defective accumulation of cyclin E may be responsible for the siSHP2-mediated cell cycle delay. To test this hypothesis, we generated a cell line that overexpressed FLAG–cyclin E. Although the level of recombinant cyclin E was also reduced by siSHP2, it was still markedly higher than the endogenous protein (Fig. 5F). Significantly, the reduction of BrdU incorporation by siSHP2 was rescued by the overexpressed cyclin E (Fig. 5G).

In normal cells, treatment with CIS induced an accumulation of cyclin E (Fig. S6A). In contrast, depletion of SHP2 with either siRNA (Fig. S6A) or shRNA (Fig. S6B) repressed the cyclin E accumulation. A previous study reported that the stabilization of cyclin E was important for prolonged S phase arrest [33]. It is conceivable that the lack of cyclin E accumulation in SHP2-depleted cells may compromise the S phase arrest, leading to premature firing of the replication origins and cell death. To test this, sensitivity to CIS was determined after cyclin E was downregulated with siRNA (Fig. S6C). In contrast to SHP2 depletion, cyclin E depletion did not result in an increase in apoptosis following CIS treatment.

Taken together, these results indicated that depletion of SHP2 lead to a reduction of cyclin E and an extension of G_1_ phase. These cell cycle defects, however, were not responsible for the increase in cell death after CIS treatment in SHP2-depleted cells.

### CHK1 Activation and the G_2_ DNA Damage Checkpoint are Impeded in the Absence of SHP2

**Figure 6:**
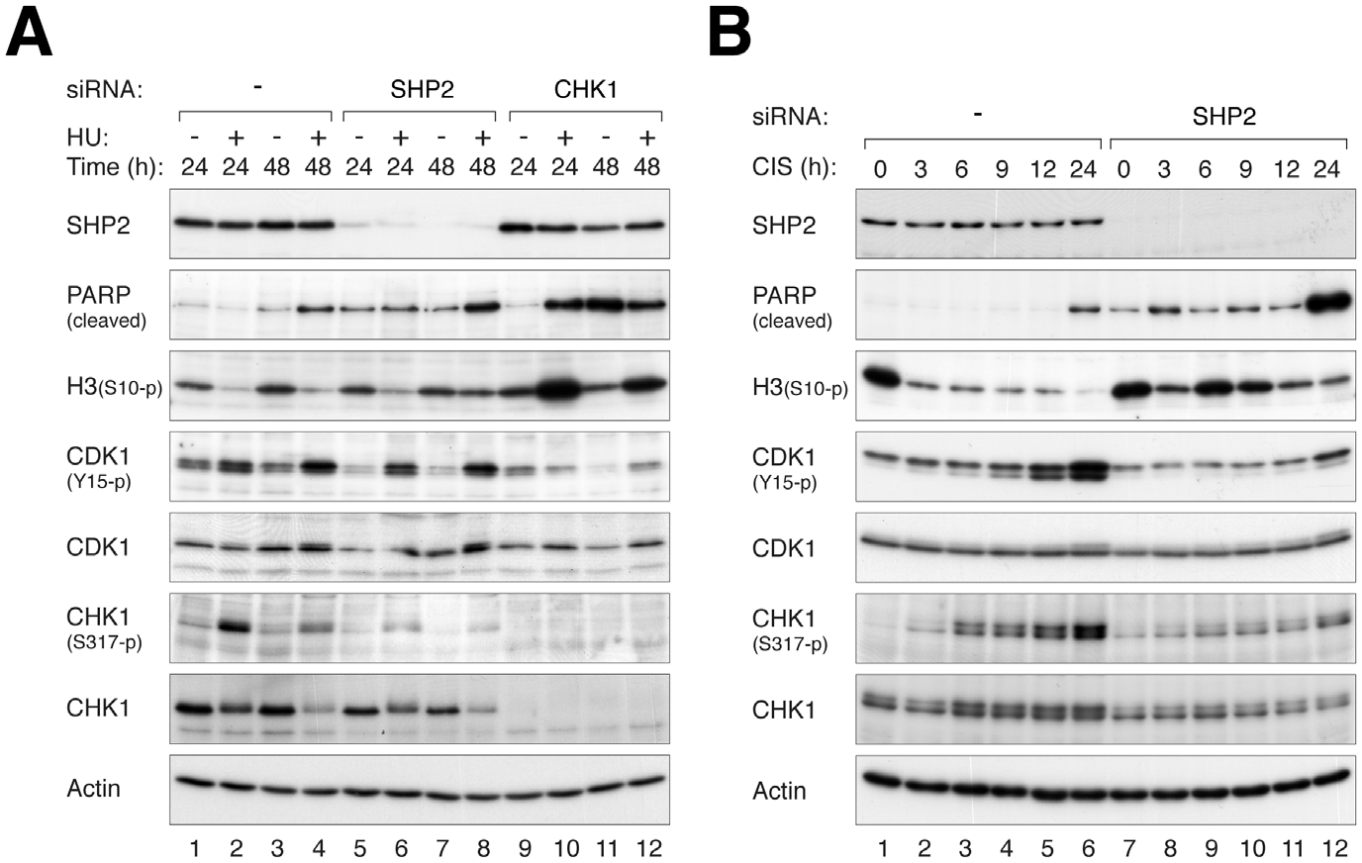
CHK1 activation is compromised in the absence of SHP2. (**A**) HU-induced CHK1 activation is dependent on SHP2. HeLa cells were transfected with control, siSHP2, or siCHK1. The cells were treated with HU and harvested at different time points. Lysates were prepared and analyzed with immunoblotting. (**B**) CIS-induced CHK1 activation is dependent on SHP2. Control- or SHP2 siRNA-transfected HeLa cells were treated with CIS and harvested at different time points. Lysates were prepared and analyzed with immunoblotting.

To investigate the integrity of the checkpoint after SHP2 depletion, we monitored the phosphorylation status of CHK1^Ser317^ after stress. As expected, HU treatment stimulated CHK1^Ser317^ phosphorylation, concomitant with the inhibition of mitotic entry (increase in CDK1^Tyr15^ and decrease in histone H3^Ser10^ phosphorylation) (Fig. 6A). The increase of CHK1^Ser317^ phosphorylation was transient (reduced at 48 h), possibly due to adaptation. Significantly, SHP2 depletion mitigated the increase in CHK1^Ser317^ phosphorylation. Likewise, the increase in phosphorylation both CHK1^Ser317^ and CDK1^Tyr15^ after CIS treatment was abolished after SHP2 depletion (Fig. 6B). The reduction of histone H3^Ser10^ phosphorylation after CIS treatment was also impaired after SHP2 depletion, suggesting that the checkpoint was bypassed without SHP2. Collectively, these data indicated that activation of the CHK1-dependent checkpoint pathway after replicative stress was dependent on SHP2.

**Figure 7:**
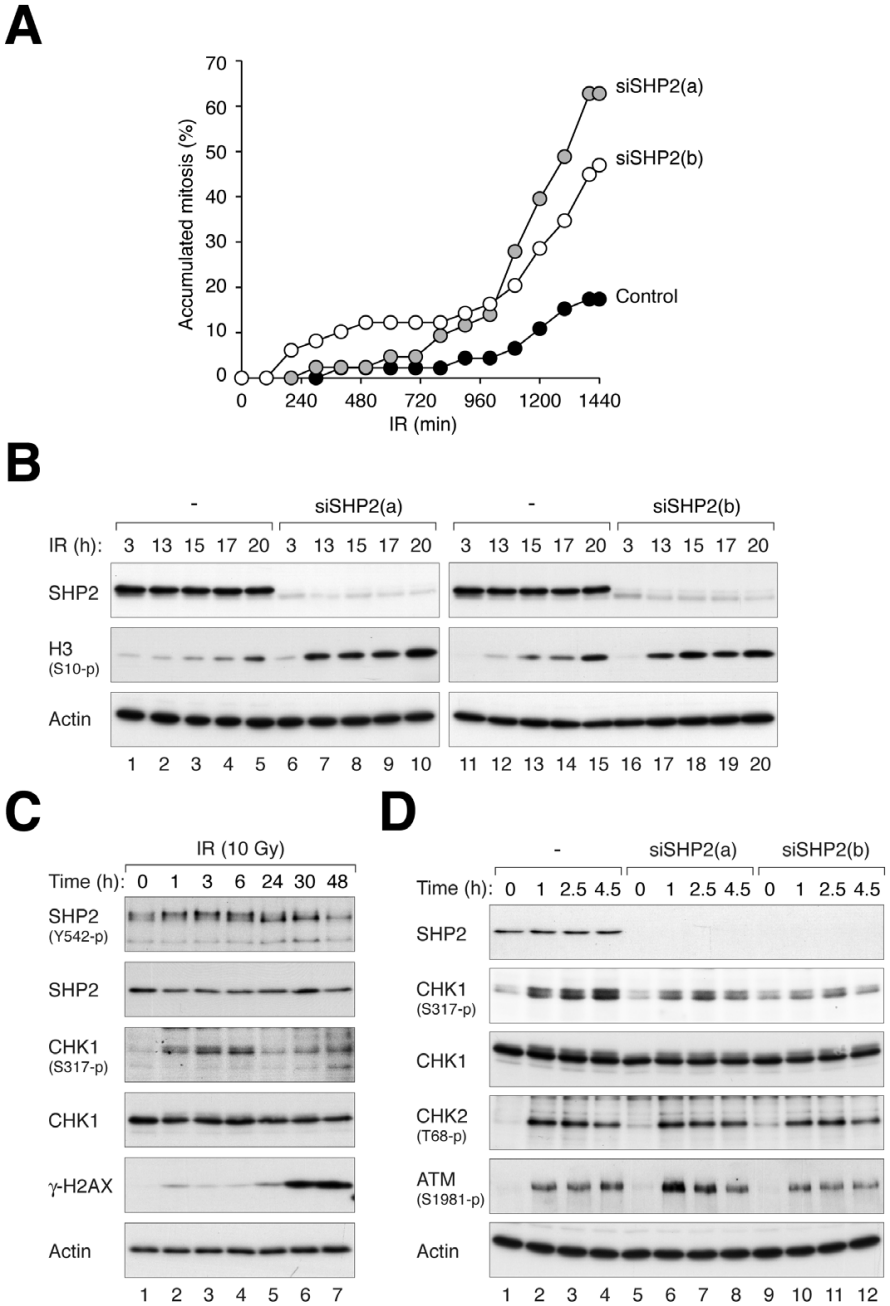
SHP2 is involved in preventing mitotic entry after DNA damage. (**A**) SHP2 is involved in maintaining the G_2_ DNA damage checkpoint. HeLa cells (expressing histone H2B–GFP) transfected with either control or SHP2 siRNA were irradiated with 10 Gy of IR. The time of mitotic entry of individual cell was tracked with time-lapse microscopy (*n* = 43–49). The data of the individual cells can be found in Fig. S7A. (**B**) SHP2 is involved in maintaining the G_2_ DNA damage checkpoint. Cells were treated as in panel B and harvested at different time points. Lysates were prepared and analyzed with immunoblotting to confirm the knockdown of SHP2. The phosphorylation of histone H3^Ser10^ indicated that cells transfected with siSHP2 were able to enter mitosis after IR treatment. (**C**) SHP2 is phosphorylated after DNA damage. HeLa cells were irradiated with 10 Gy of IR and harvested at the indicated time points. The cell pellets were directly boiled in sample buffer and analyzed with immunoblotting. (**D**) IR-induced CHK1 activation is impaired in SHP2-depleted cells. Control, siSHP2(a)-, or siSHP2(b)-transfected cells were treated with 10 Gy of IR and harvested at the indicated time points. Lysates were prepared and analyzed with immunoblotting.

Given that CIS and HU ultimately induced both replication block and DNA damage, we next examined if SHP2 is also involved in DNA damage responses. Double-strand breaks were introduced with ionizing radiation (IR), which mainly activates the G_2_ DNA damage checkpoint in HeLa cells. Time-lapse microscopy indicated that cells were prevented from entering mitosis by IR. Some recovery could be seen after 12 h, probably due to either repair or adaptation (Fig. 7A). In contrast, significantly more SHP2-depleted cells were able to enter mitosis after irradiation, indicating defective checkpoint responses. Consistent with these results, SHP2 depletion also abolished the IR-mediated suppression of histone H3^Ser10^ phosphorylation (Fig. 7B).

The G_2_ DNA damage checkpoint is regulated by both the classic ATM/ATR–CHK1/CHK2 axis as well as a pathway involving the MAPK p38 (ATM/ATR–p38–MK2) [34], [35]. Since SHP2 acts on the MAPK ERK in growth regulation, it is conceivable that the role of SHP2 in the G_2_ DNA damage checkpoint may also involve MAPK. However, IR-mediated interphase arrest was insensitive to inhibitors of p38 (SB203580) or ERK (U0126, a MEK1/MEK2 inhibitor) (Fig. S7B). By contrast, the checkpoint was readily uncoupled by the CHK1 inhibitor UCN-01, suggesting that the effects of SHP2 depletion on the checkpoint was unlikely to be due to a decrease of p38 or ERK activity.

Further evidence of a role of SHP2 in the DNA damage checkpoint was seen by the activation of SHP2 (Tyr542 phosphorylation) after DNA damage (Fig. 7C). Interestingly, IR also induced a gel mobility shift of SHP2, which was independent on Tyr542 phosphorylation and occurred at a similar kinetics as the activation of CHK1. Importantly, the IR-mediated phosphorylation of CHK1^Ser317^ was reduced after SHP2 was depleted with siRNA (Fig. 7D).

**Figure 8:**
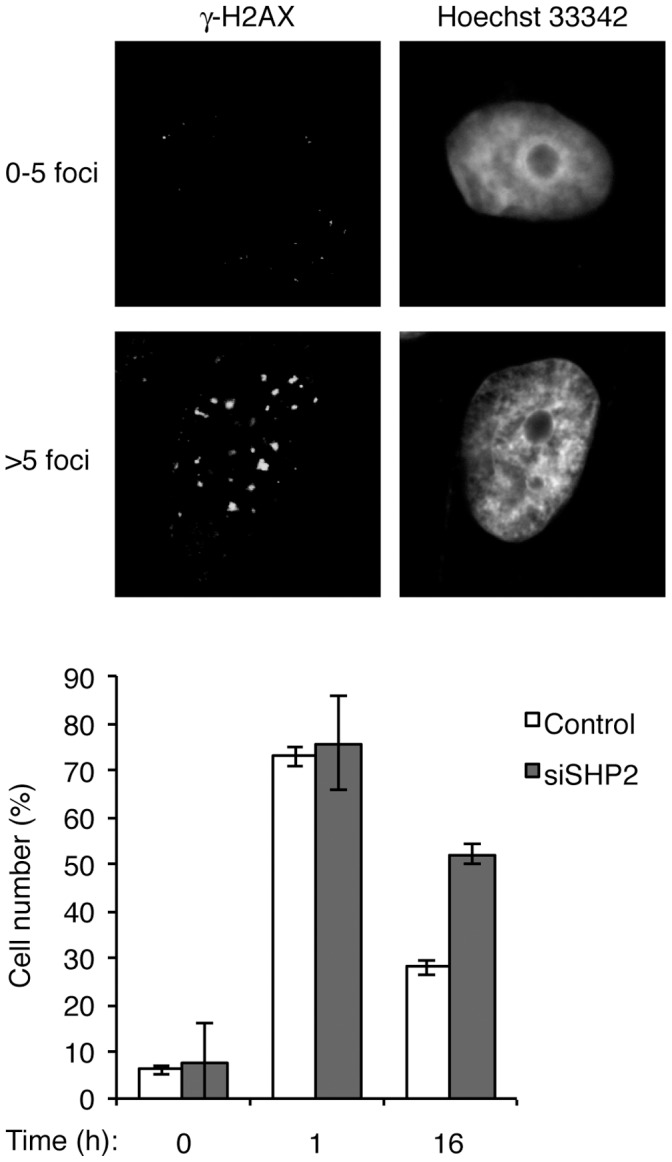
SHP2 is required for efficient repair of DNA damage. Mock- or SHP2-depleted HeLa cells were either untreated (t = 0 h) or irradiated with 10 Gy of IR. At different time points, the cells were fixed and stained for γ-H2AX. The number of cells with more than five γ-H2AX foci was quantified (*n* = 50; Mean±SD of three independent experiments). Representative images of the γ-H2AX staining are shown on the top.

As defective CHK1 activation results in inefficient DNA repair [36], we next evaluated if repair was also compromised in the absence of SHP2. In control cells, the number of γ-H2AX foci increased shortly after IR treatment (1 h). Reduction of the number of γ-H2AX foci at later time points (16 h) indicated that some damage sites were repaired (Fig. 8). In contrast, the number of γ-H2AX foci sustained at a higher level in SHP2-depleted cells, suggesting a deficiency of DNA repair in these cells. A similar defect of DNA repair was also detected when SHP2-depleted cells were damaged with CIS (Fig. S8).

Collectively, these results indicated that SHP2 was essential for the full activation of CHK1, checkpoint-mediated cell cycle arrest, and DNA repair in response to DNA damage.

## Discussion

Using a RNAi-mediated screen of the phosphatome, we have identified several phosphatases that were essential for cell survival after HU challenge (Fig. 1B). As with other similar screening approaches, potential candidates may not be identified due to various reasons, including incomplete depletion and functional redundancy. Caveats are also required for the interpretations of the positive candidates. Although not all the candidates have been characterized in detail, it is likely that not all the RNAi effects were specific. In fact, the mRNA of some of the candidates were not effectively depleted by the shRNAs (Fig. 1C). Nevertheless, our data indicated the ability of the assay to identify physiological-relevant candidates. Depletion of the classic checkpoint component, CHK1, was used as a positive control (Fig. S1). Moreover, one of the positive clones, dual specificity phosphatase 11 (DUSP11), has already been implicated in the G_2_ DNA damage checkpoint [37]. It was found that U2OS cells that expressed DUSP11 shRNA retained histone H3^Ser10^ phosphorylation after irradiation.

Our data indicated that downregulation of SHP2 with either siRNA or shRNA, both with transient transfection and in stable cell lines, enhanced HU-mediated cell death (Fig. 1 and Fig. 2). SHP2 was also required for cell survival after challenge with other DNA stress-inducing agents, including CIS (Fig. 6) and IR (Fig. 7). Unlike HU, CIS could generate inter-and intra-strand crosslinks, causing single and double-strand breaks in addition to replication blockage. This may explain the relatively high level of cell death associated with CIS. Consistent with these results, we found that SHP2 was activated (as indicated by SHP2^Tyr542^ phosphorylation) upon treatment with HU (Fig. 3A), CIS (Fig. 3B), and IR (Fig. 7C). In agreement with our results, Kuo *et al*. recently demonstrated using an activity-based probe that SHP2 is activated by CIS [31]. The specificity of the effects on SHP2 was demonstrated with a number of approaches, including rescue experiments using shSHP2-resistant SHP2 (Fig. 4F) as well as using a small inhibitor of SHP2 (Fig. 4G).

SHP2 has mainly been implicated in cell growth regulation. Indeed, depletion of SHP2 extended the cell cycle by ∼2 h in HeLa cells (Fig. 5A). We found that the cell cycle delay was probably caused by a reduction of cyclin E and an extension of G_1_ phase. However, the cell cycle defects were not responsible for the increase in cell death after CIS treatment in SHP2-depleted cells (Fig. 5).

We found that the increase in cell death after SHP2 downregulation was at least in part due to defective checkpoint mechanisms. The activation of CHK1 after HU (Fig. 6A), CIS (Fig. 6B), and IR (Fig. 7D) treatment was reduced after SHP2 depletion. Consistent with the weakening of the checkpoint, the reduction of histone H3^Ser10^ phosphorylation after DNA damage was prevented in SHP2-depleted cells (Fig. 6 and Fig. 7D). Indeed, SHP2-depleted cells prematurely entered mitosis after IR treatment (Fig. 7A). Another indication of the weakening of the checkpoint after SHP2 depletion was the impairment of DNA repair (Fig. 8 and Fig. S8). The premature entry into mitosis of HU- or CIS-treated S phase cells probably resulted directly in apoptosis (Fig. 2 and Fig. 4). In accordance with our findings, the IR-mediated G_2_ arrest response was found to be diminished in MEFs lacking functional Shp2 [38]. It should be noted, however, SHP2 probably only plays a peripheral role in the checkpoint. Depletion of the classic checkpoint component CHK1 induced significantly more checkpoint defects than SHP2 (Fig. 2A).

As SHP2 is implicated in the regulation of several proteins that can potentially regulate apoptosis (including AKT, ERK, and JNK), it is perhaps not surprising that depletion of SHP2 affected cell survival in our studies. However, previously published evidence of SHP2’s effects on apoptosis is contentious. Consistent with our findings, Shp2-deficient mouse embryonic fibroblasts (MEFs) are hypersensitive to etoposide- or proteasome inhibitor MG115-induced apoptosis [39], [40]. Likewise, inactivation of Shp2 sensitizes MEFs to epigallocatechin-3-gallate (green tea polyphenol)-mediated apoptosis through p53- and p73-dependent mechanisms [41]. Murine hemopoietic cell line Ba/F3 overexpressing catalytically inactive Shp2 are also more susceptible to cell death [42]. However, contrary to our findings, Shp2-deficient MEFs were found to be less sensitive to CIS-induced apoptosis [43]. Shp2 has also been reported to negatively regulate hematopoietic cell survival by dephosphorylation of Stat5 [44]. It should be noted that the studies using Shp2-deficent cells involves the use of immortalized MEFs with a deletion within exon 3 of Shp2, which results in a truncation that lacks the NH_2_-terminal SH2 domain (N-SH2), but retains catalytic activity [45], [46]. The effects of the Shp2 truncation in MEFs may differ from the depletion of the entire SHP2 in this study. In accordance with this, Shp2^−/−^ mice died at peri-implantation, much earlier than Shp2^exon 3−/−^ mice (midgestation) [47]. In agreement with our results, RNAi-mediated knockdown of Shp2 in murine myoblast C2C12 also reduces cell survival [39].

In conclusion, SHP2 is both activated upon several DNA stress signals and is required for maintaining the checkpoint. The depletion of SHP2 resulted in defects in DNA damage-induced CHK1 activation, cell cycle arrest, and DNA repair, thereby promoting apoptosis.

## Supporting Information

Figure S1 **Screening of phosphatases that are important for the DNA replication checkpoint. (A)** Schematic diagram of the shRNA library screening. **(B)** Depletion of CHK1 as a positive control for the HU-mediated checkpoint screens. HeLa cells were transfected with control vector or plasmids expressing CHK1 shRNA. A plasmid expressing histone H2B–GFP was co-transfected. At 48 h after transfection, the cells were incubated with HU for another 48 h. Representative fluorescence microscopy images indicate that while HU arrested the cell cycle in vector-transfected cells (cells displayed no mitosis and contained a larger nucleus), massive cell death was induced in shCHK1-transfected cells. **(C)** Depletion of CHK1 induces massive cell death in HU-treated cells. Cells were transfected and treated as in panel B. The percentage of cell death was quantified (*n* = 200). Mean±SD of three independent experiments is shown. **(D)** Depletion of CHK1 bypasses the HU-mediated checkpoint. Cells were transfected and treated as in panel B and harvested at 24 h and 48 h. Lysates were prepared and the indicated proteins were detected with immunoblotting. Note that the CHK1 band appears weaker after HU treatment due to multiple phosphorylation-mediated gel mobility shifts. Note also that depletion of CHK1 was incomplete because both transfected and non-transfected cells were harvested together. Actin analysis was included to assess protein loading and transfer.
(PDF)

Figure S2 **Downregulation of SHP2 enhances CIS-mediated cell death. (A)** Downregulation of SHP2 sensitizes cells to CIS. HeLa cells transfected with control, SHP2, or CHK1 siRNAs were treated with either buffer or CIS. After 24 h, cell death was analyzed with fluorescence microscopy (*n* = 400). Representative images are shown. **(B)** Time-lapse imaging reveals that downregulation of SHP2 enhances CIS-mediated cell death. HeLa cells stably expressing histone H2B–GFP were transfected with either control or siSHP2. After treatment with CIS, the fate of individual cells were tracked with time-lapse microscopy at 5 min/frame for 24 h (*n* = 100). Each horizontal line represents one cell. Key: light grey = interphase; black = mitosis (from DNA condensation to anaphase or cell death); truncated bars = cell death.
(PDF)

Figure S3 **Depletion of SHP2 enhances CIS-mediated cell death. (A)** Depletion of SHP2 enhances CIS-mediated cell death in Hep3B cells. Hep3B cells transfected with control or siSHP2 were treated with either buffer or CIS. After 24 h, the cells were harvested and analyzed with flow cytometry. **(B)** shSHP2-expressing stable cell lines are hypersensitive to CIS. Three clones of shRNA-expressing HeLa cells were treated with buffer or CIS. After 24 h, the cells were harvested and analyzed with flow cytometry. The expression of SHP2 was confirmed with immunoblotting.
(PDF)

Figure S4 **Cisplatin induces cell death in SHP2-depleted cells by apoptosis. (A)** Inhibition of caspases abolishes CIS-induced sub-G_1_ population in SHP2-depleted cells. HeLa cells transfected with control, siSHP2, or siCHK1 were treated with a combination of CIS, caspase inhibitor (CI), and nocodazole (NOC). After 24 h, the cells were harvested and analyzed with flow cytometry. The percentage of sub-G_1_ population is indicated in each panel. **(B)** Inhibition of caspases abolishes CIS-induced PARP cleavage in SHP2-depleted cells. Cells were treated as in panel A. After 24 h, the cells were harvested and analyzed with immunoblotting. **(C)** Expression of BCL2 abolishes siSHP2-mediated cell death. HeLa or HeLa overexpressing FLAG–BCL2 were transfected with either control or siSHP2, before treated with buffer or CIS. After 24 h, the cells were harvested and analyzed with flow cytometry. The percentage of sub-G_1_ population is indicated in each panel. **(D)** Expression of BCL2 abolishes siSHP2-mediated PARP cleavage. Cells were treated as in panel C. Lysates were prepared and analyzed with immunoblotting to confirm the knockdown of SHP2 and expression of FLAG–BCL2 (the faint band below FLAG-BCL2 is from the previous SHP2 blot). Apoptosis was analyzed with antibodies that specifically recognizes cleaved PARP.
(PDF)

Figure S5 **Depletion of both SHP2 delays cell cycle progression. (A)** Depletion of SHP2 in HeLa cells lengthens the cell cycle by ∼2 h. HeLa cells expressing histone H2B–GFP were transfected with either control (*n* = 37) or SHP2 siRNA (*n* = 23). Time-lapse microscopy was used to track individual cells for 30 h. The time of entry into the first mitosis to the end of the second mitosis of individual cells is plotted. Each horizontal line represents one cell. Key: light grey = interphase; black = mitosis (from DNA condensation to anaphase or cell death). **(B)** Depletion of both SHP2 and cyclin E delays cell cycle progression. HeLa cells transfected with control, SHP2, or cyclin E siRNA were treated with nocodazole. The cells were harvested for flow cytometry analysis at the indicated time points.
(PDF)

Figure S6 **CIS-mediated cyclin E accumulation is dependent on SHP2. (A)** SHP2 siRNA abolishes CIS-induced cyclin E accumulation. Control and siSHP2-transfected cells were treated with CIS. At the indicated time points, lysates were prepared and analyzed with immunoblotting. **(B)** SHP2 shRNA abolishes CIS-induced cyclin E accumulation. HeLa cells were transfected with plasmids expressing control or SHP2 shRNA. A plasmid expressing a blasticidin-resistant gene was co-transfected. Transfected cells were enriched by blasticidin treatment for 36 h and allowed to recover for 24 h. The cells were then treated with CIS and harvested at different time for immunoblotting analysis. **(C)** The increase of cell death in SHP2-depleted cells is independent on the defects on cyclin E accumulation. HeLa cells were transfected with control, siSHP2(a), siSHP2(b), or cyclin E siRNA. The cells were treated with either buffer or CIS for 24 h. The cells were then harvested either for flow cytometry analysis (the levels of sub-G_1_ cells were quantified) or immunoblotting to confirm the knockdown (bottom panel).
(PDF)

Figure S7 **Depletion of SHP2 disrupts the IR-induced DNA damage checkpoint. (A)** HeLa cells (expressing histone H2B–GFP) transfected with either control or SHP2 siRNA were irradiated with 10 Gy of IR. The fates of individual cells were tracked with time-lapse microscopy. Each horizontal line represents one cell. Key: light grey = interphase; black = mitosis (from DNA condensation to anaphase or cell death); truncated bars = cell death. **(B)** IR-mediated arrest is insensitive to inhibition of p38 and ERK. HeLa cells expressing histone H2B–GFP were incubated with SB203580 (p38 inhibitor), U0126 (MEK1/2 inhibitor), or UCN-01 (CHK1 inhibitor) at 1 h prior to IR treatment. After irradiation (10 Gy), the cells were tracked with time-lapse microscopy for 24 h. Each horizontal line represents one cell. Key: light grey = interphase; black = mitosis (from DNA condensation to anaphase or cell death); truncated bars = cell death. Quantification of the accumulative percentage of mitosis is shown.
(PDF)

Figure S8 **Cells lacking SHP2 are more susceptible to CIS-mediated DNA damage. (A)** DNA repair is less effective in SHP2-depleted cells. HeLa cells transfected with control or SHP2 siRNA were treated with CIS. A caspase inhibitor was included to prevent cell death. After 24 h, the cells were fixed and stained for γ-H2AX. Representative images of the γ-H2AX staining are shown. **(B)** The number of γ-H2AX foci in cells treated in panel A was quantified (*n* = 50). Mean±SD of three independent experiments is shown. **(C)** Cells were treated as in panel A. Lysates were prepared and the knockdown of SHP2 was confirmed by immunoblotting.
(PDF)

Table S1 **List of clones in the phosphatome library.** The gene symbol, name, and accession number of the genes represented in the library are indicated. The sequences of the two shRNAs for each gene and the z-scores of the hydroxyurea screen are also shown.
(PDF)
