# Supplementary figures and images for: Novel Functions of the Phosphatase SHP2 in the DNA Replication and Damage Checkpoints
Source: PLoS One. 2012 Nov 26;7(11):e49943. doi: 10.1371/journal.pone.0049943 (PMC3506573; doi:10.1371/journal.pone.0049943)

**A**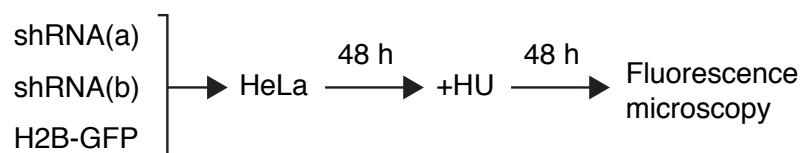**B**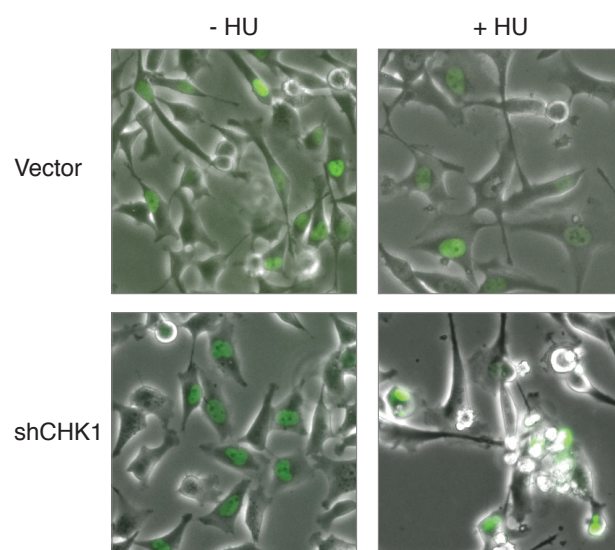**C**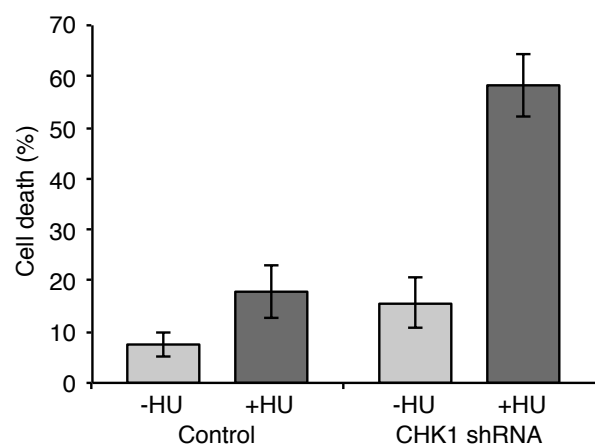**D**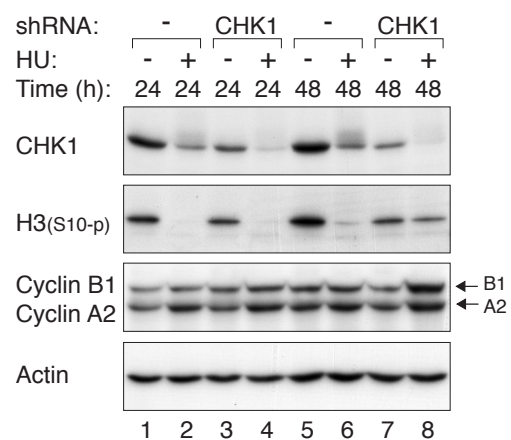

Supplement: Figure S1 — Screening of phosphatases that are important for the DNA replication checkpoint. (A) Schematic diagram of the shRNA library screening. (B) Depletion of CHK1 as a positive control for the HU-mediated checkpoint screens. HeLa cells were transfected with control vector or plasmids expressing CHK1 shRNA. A plasmid expressing histone H2B–GFP was co-transfected. At 48 h after transfection, the cells were incubated with HU for another 48 h. Representative fluorescence microscopy images indicate that while HU arrested the cell cycle in vector-transfected cells (cells displayed no mitosis and contained a larger nucleus), massive cell death was induced in shCHK1-transfected cells. (C) Depletion of CHK1 induces massive cell death in HU-treated cells. Cells were transfected and treated as in panel B. The percentage of cell death was quantified (n = 200). Mean±SD of three independent experiments is shown. (D) Depletion of CHK1 bypasses the HU-mediated checkpoint. Cells were transfected and treated as in panel B and harvested at 24 h and 48 h. Lysates were prepared and the indicated proteins were detected with immunoblotting. Note that the CHK1 band appears weaker after HU treatment due to multiple phosphorylation-mediated gel mobility shifts. Note also that depletion of CHK1 was incomplete because both transfected and non-transfected cells were harvested together. Actin analysis was included to assess protein loading and transfer. (PDF) [file pone.0049943.s001.pdf]

**A**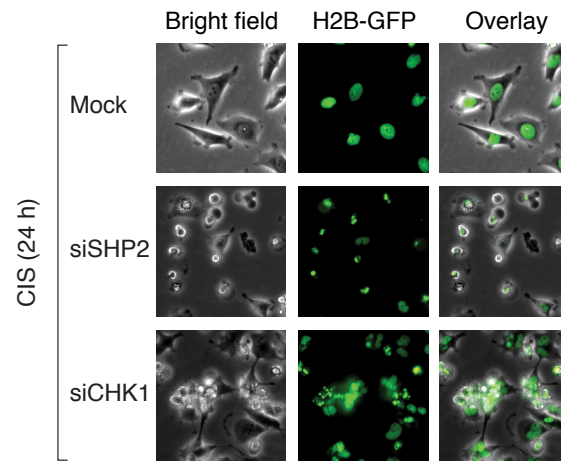**B**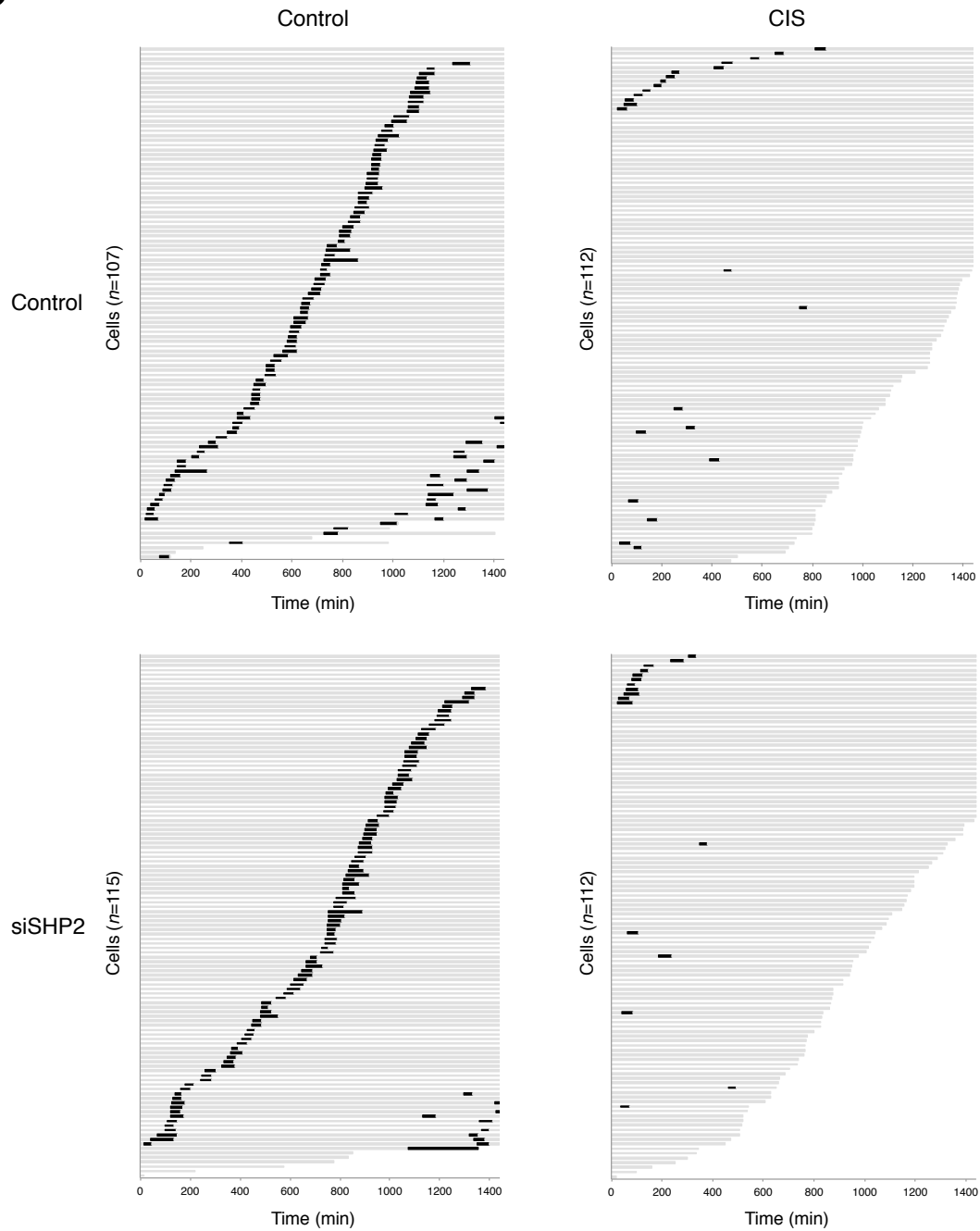

Supplemental Figure S2

Supplement: Figure S2 — Downregulation of SHP2 enhances CIS-mediated cell death. (A) Downregulation of SHP2 sensitizes cells to CIS. HeLa cells transfected with control, SHP2, or CHK1 siRNAs were treated with either buffer or CIS. After 24 h, cell death was analyzed with fluorescence microscopy (n = 400). Representative images are shown. (B) Time-lapse imaging reveals that downregulation of SHP2 enhances CIS-mediated cell death. HeLa cells stably expressing histone H2B–GFP were transfected with either control or siSHP2. After treatment with CIS, the fate of individual cells were tracked with time-lapse microscopy at 5 min/frame for 24 h (n = 100). Each horizontal line represents one cell. Key: light grey = interphase; black = mitosis (from DNA condensation to anaphase or cell death); truncated bars = cell death. (PDF) [file pone.0049943.s002.pdf]

**A**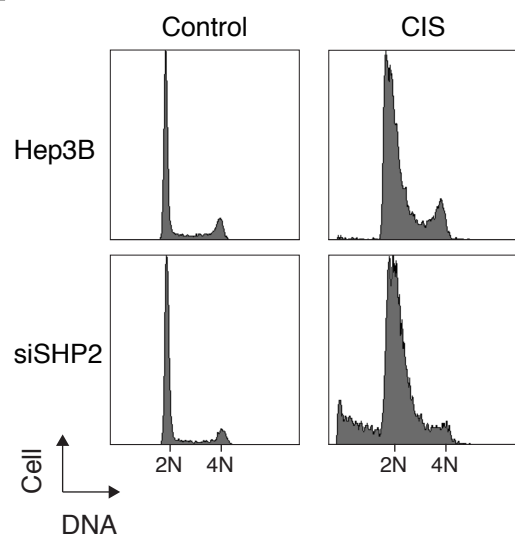**B**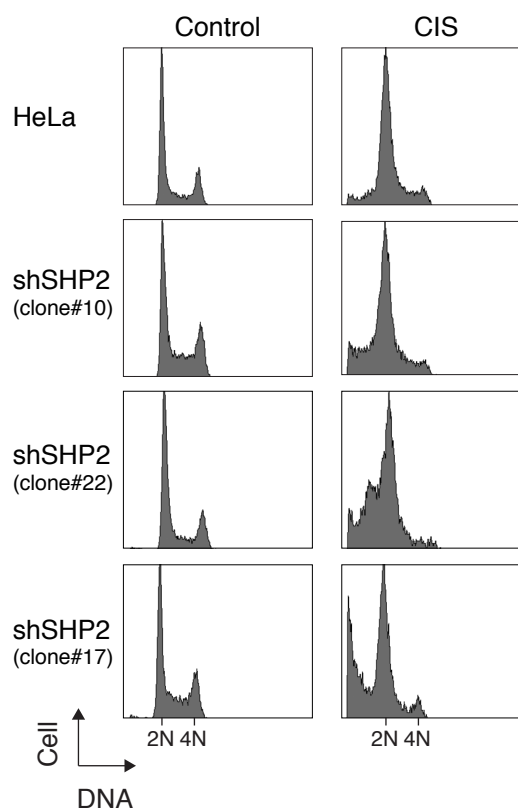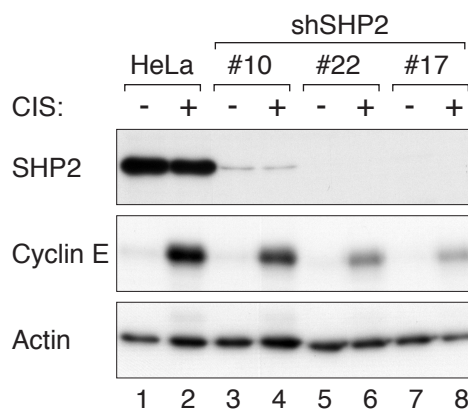

Supplement: Figure S3 — Depletion of SHP2 enhances CIS-mediated cell death. (A) Depletion of SHP2 enhances CIS-mediated cell death in Hep3B cells. Hep3B cells transfected with control or siSHP2 were treated with either buffer or CIS. After 24 h, the cells were harvested and analyzed with flow cytometry. (B) shSHP2-expressing stable cell lines are hypersensitive to CIS. Three clones of shRNA-expressing HeLa cells were treated with buffer or CIS. After 24 h, the cells were harvested and analyzed with flow cytometry. The expression of SHP2 was confirmed with immunoblotting. (PDF) [file pone.0049943.s003.pdf]

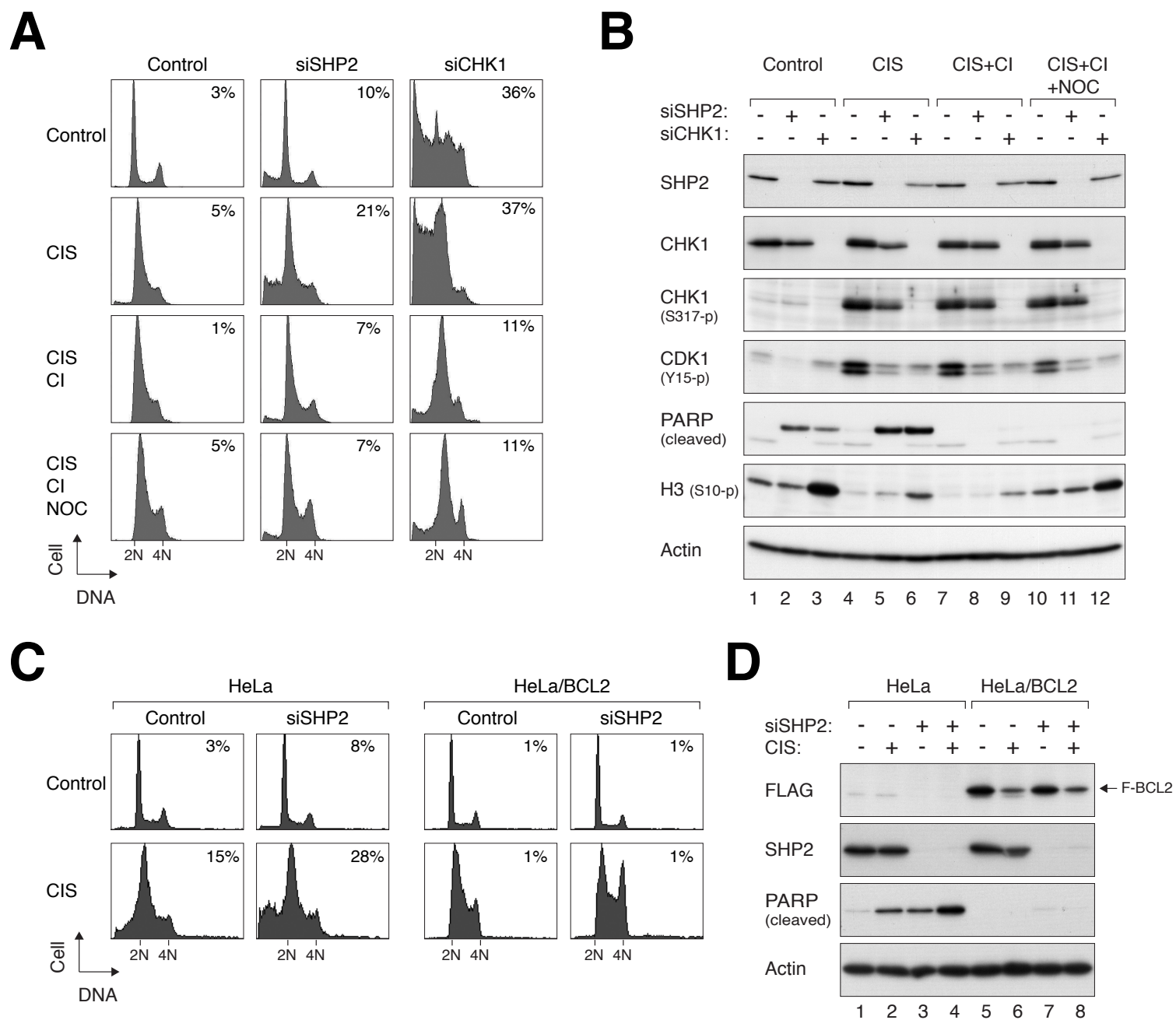

Supplemental Figure S4

Supplement: Figure S4 — Cisplatin induces cell death in SHP2-depleted cells by apoptosis. (A) Inhibition of caspases abolishes CIS-induced sub-G1 population in SHP2-depleted cells. HeLa cells transfected with control, siSHP2, or siCHK1 were treated with a combination of CIS, caspase inhibitor (CI), and nocodazole (NOC). After 24 h, the cells were harvested and analyzed with flow cytometry. The percentage of sub-G1 population is indicated in each panel. (B) Inhibition of caspases abolishes CIS-induced PARP cleavage in SHP2-depleted cells. Cells were treated as in panel A. After 24 h, the cells were harvested and analyzed with immunoblotting. (C) Expression of BCL2 abolishes siSHP2-mediated cell death. HeLa or HeLa overexpressing FLAG–BCL2 were transfected with either control or siSHP2, before treated with buffer or CIS. After 24 h, the cells were harvested and analyzed with flow cytometry. The percentage of sub-G1 population is indicated in each panel. (D) Expression of BCL2 abolishes siSHP2-mediated PARP cleavage. Cells were treated as in panel C. Lysates were prepared and analyzed with immunoblotting to confirm the knockdown of SHP2 and expression of FLAG–BCL2 (the faint band below FLAG-BCL2 is from the previous SHP2 blot). Apoptosis was analyzed with antibodies that specifically recognizes cleaved PARP. (PDF) [file pone.0049943.s004.pdf]

**A**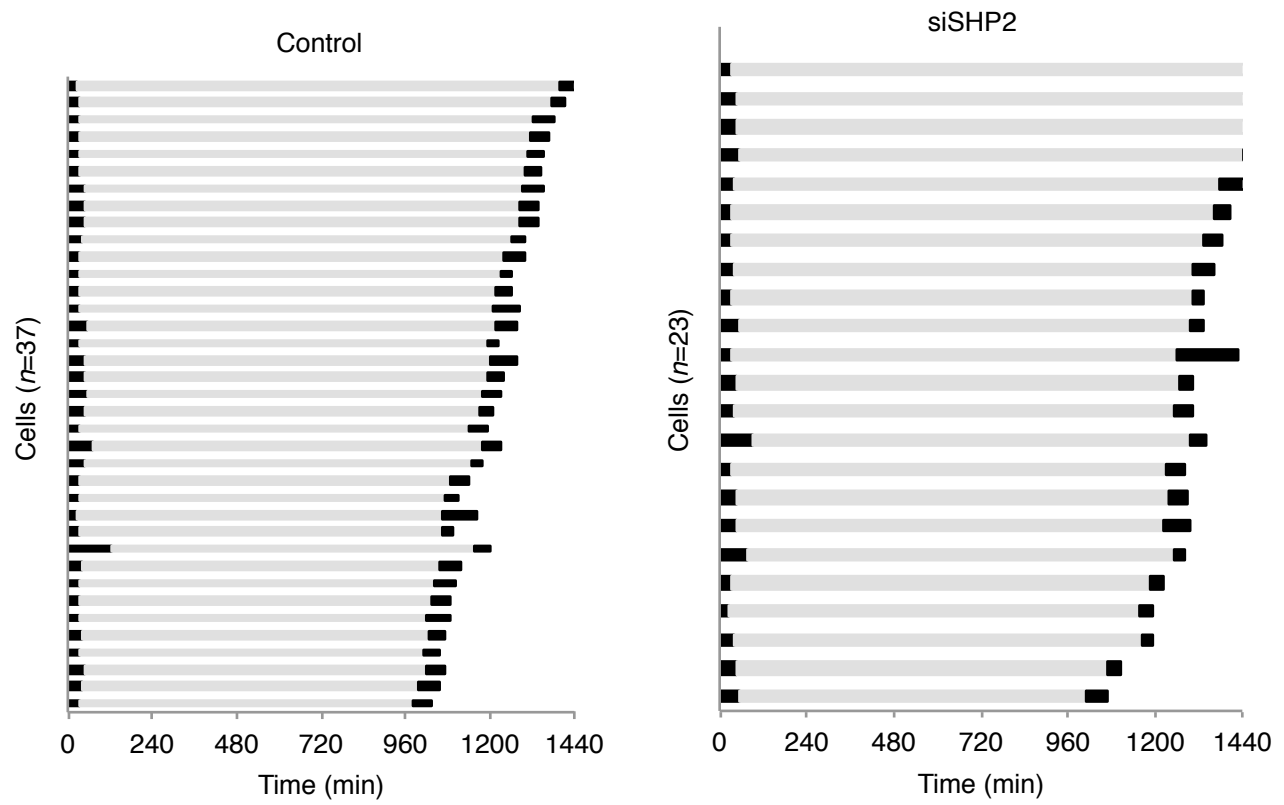**B**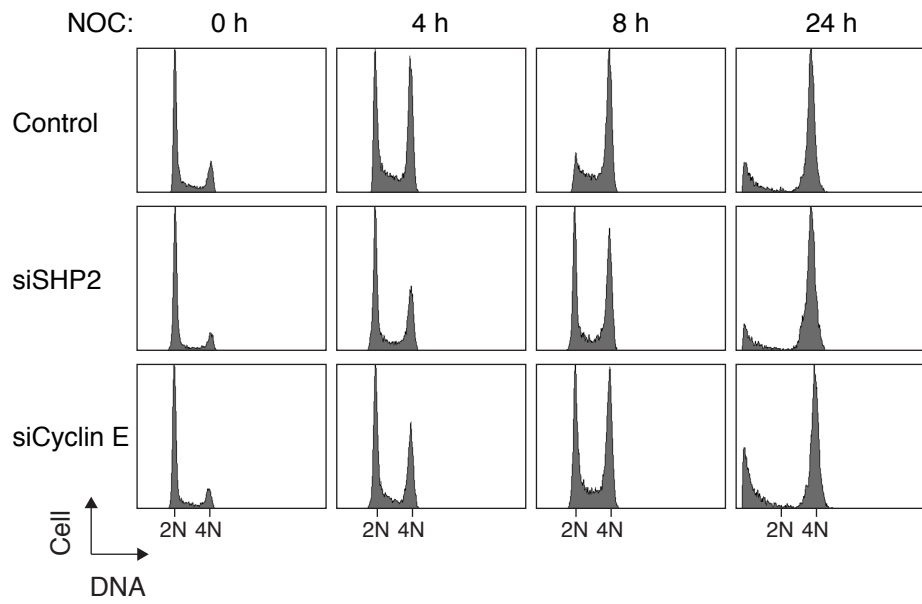

Supplement: Figure S5 — Depletion of both SHP2 delays cell cycle progression. (A) Depletion of SHP2 in HeLa cells lengthens the cell cycle by ∼2 h. HeLa cells expressing histone H2B–GFP were transfected with either control (n = 37) or SHP2 siRNA (n = 23). Time-lapse microscopy was used to track individual cells for 30 h. The time of entry into the first mitosis to the end of the second mitosis of individual cells is plotted. Each horizontal line represents one cell. Key: light grey = interphase; black = mitosis (from DNA condensation to anaphase or cell death). (B) Depletion of both SHP2 and cyclin E delays cell cycle progression. HeLa cells transfected with control, SHP2, or cyclin E siRNA were treated with nocodazole. The cells were harvested for flow cytometry analysis at the indicated time points. (PDF) [file pone.0049943.s005.pdf]

**A**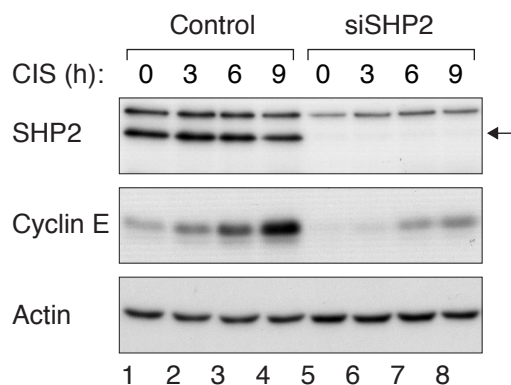**B**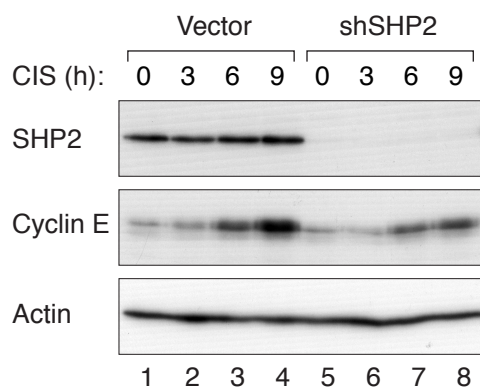**C**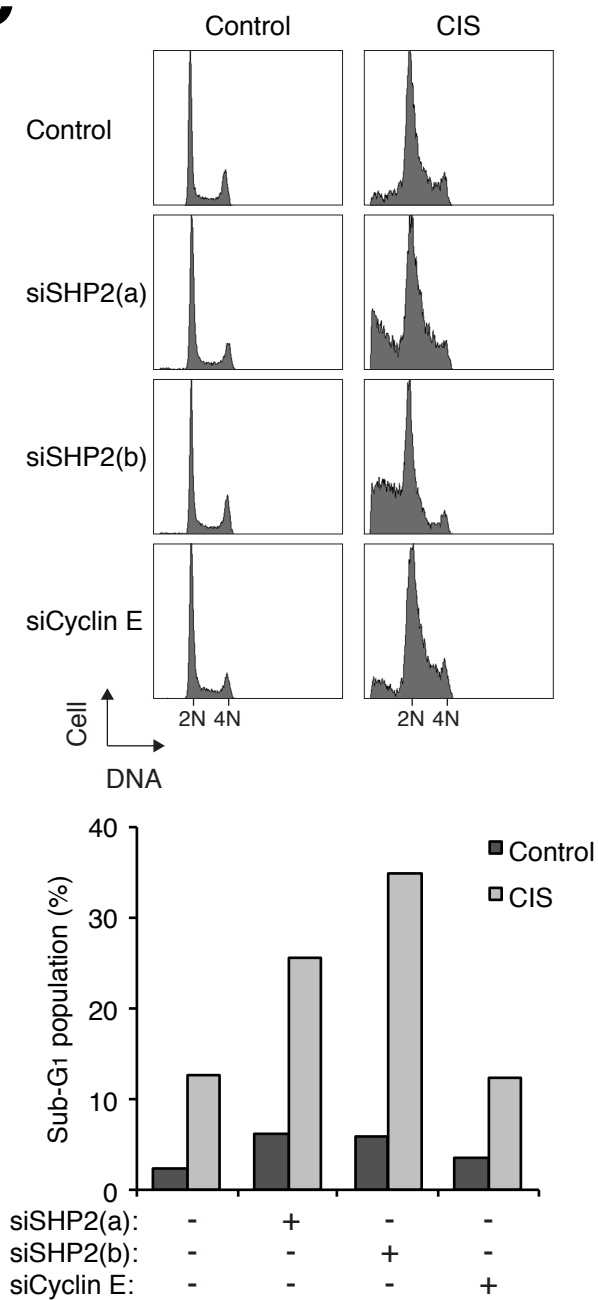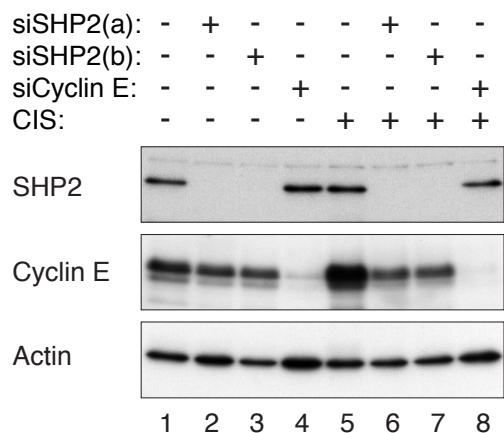

Supplement: Figure S6 — CIS-mediated cyclin E accumulation is dependent on SHP2. (A) SHP2 siRNA abolishes CIS-induced cyclin E accumulation. Control and siSHP2-transfected cells were treated with CIS. At the indicated time points, lysates were prepared and analyzed with immunoblotting. (B) SHP2 shRNA abolishes CIS-induced cyclin E accumulation. HeLa cells were transfected with plasmids expressing control or SHP2 shRNA. A plasmid expressing a blasticidin-resistant gene was co-transfected. Transfected cells were enriched by blasticidin treatment for 36 h and allowed to recover for 24 h. The cells were then treated with CIS and harvested at different time for immunoblotting analysis. (C) The increase of cell death in SHP2-depleted cells is independent on the defects on cyclin E accumulation. HeLa cells were transfected with control, siSHP2(a), siSHP2(b), or cyclin E siRNA. The cells were treated with either buffer or CIS for 24 h. The cells were then harvested either for flow cytometry analysis (the levels of sub-G1 cells were quantified) or immunoblotting to confirm the knockdown (bottom panel). (PDF) [file pone.0049943.s006.pdf]

**A**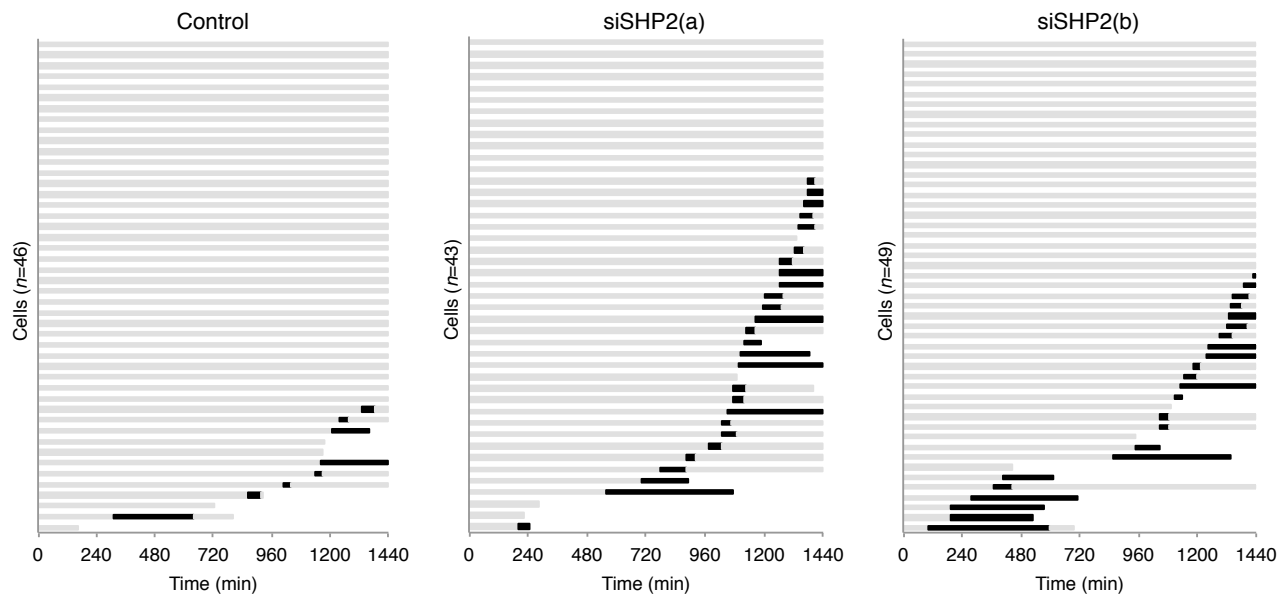**B**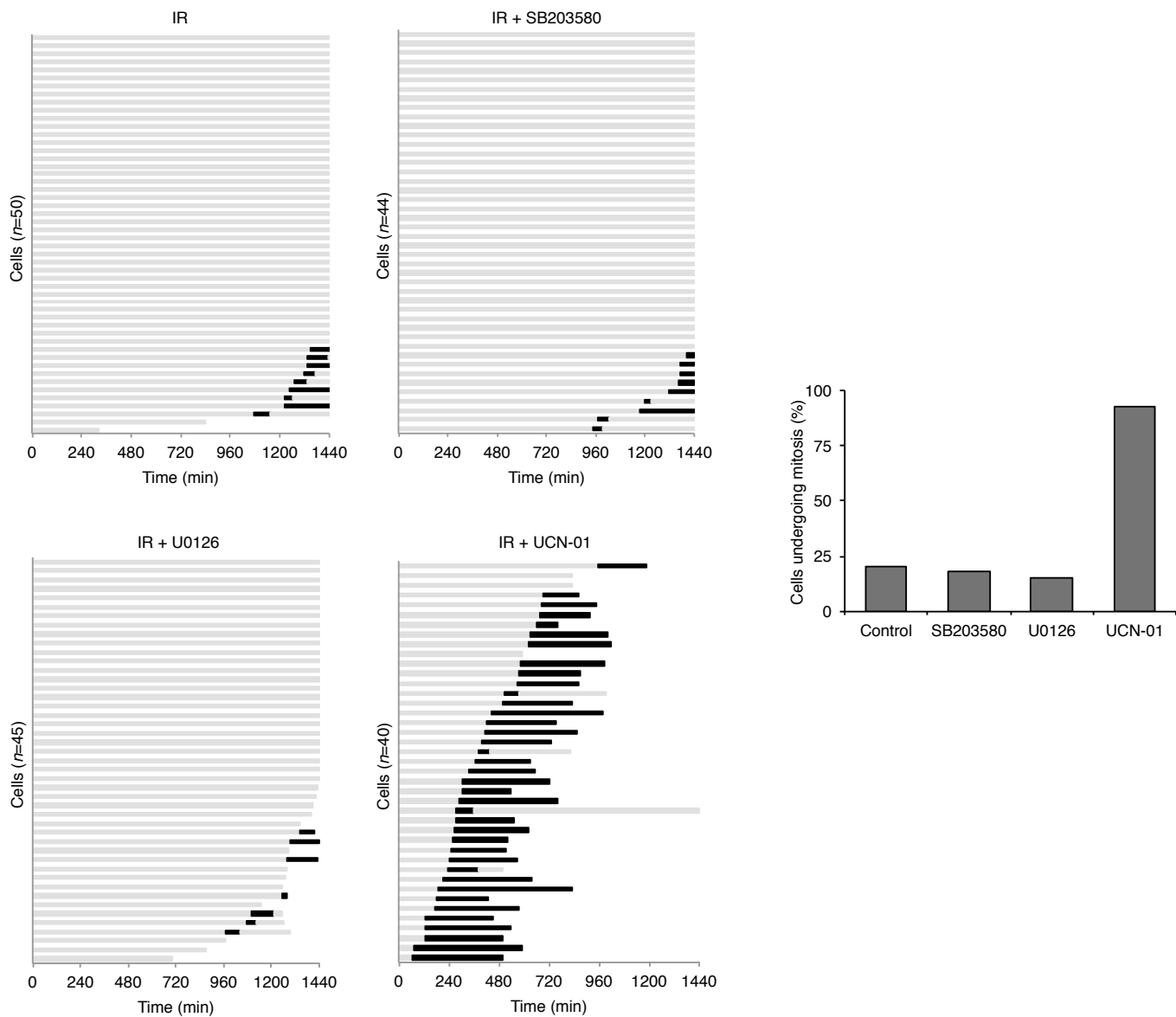

Supplemental Figure S7

Supplement: Figure S7 — Depletion of SHP2 disrupts the IR-induced DNA damage checkpoint. (A) HeLa cells (expressing histone H2B–GFP) transfected with either control or SHP2 siRNA were irradiated with 10 Gy of IR. The fates of individual cells were tracked with time-lapse microscopy. Each horizontal line represents one cell. Key: light grey = interphase; black = mitosis (from DNA condensation to anaphase or cell death); truncated bars = cell death. (B) IR-mediated arrest is insensitive to inhibition of p38 and ERK. HeLa cells expressing histone H2B–GFP were incubated with SB203580 (p38 inhibitor), U0126 (MEK1/2 inhibitor), or UCN-01 (CHK1 inhibitor) at 1 h prior to IR treatment. After irradiation (10 Gy), the cells were tracked with time-lapse microscopy for 24 h. Each horizontal line represents one cell. Key: light grey = interphase; black = mitosis (from DNA condensation to anaphase or cell death); truncated bars = cell death. Quantification of the accumulative percentage of mitosis is shown. (PDF) [file pone.0049943.s007.pdf]

# A

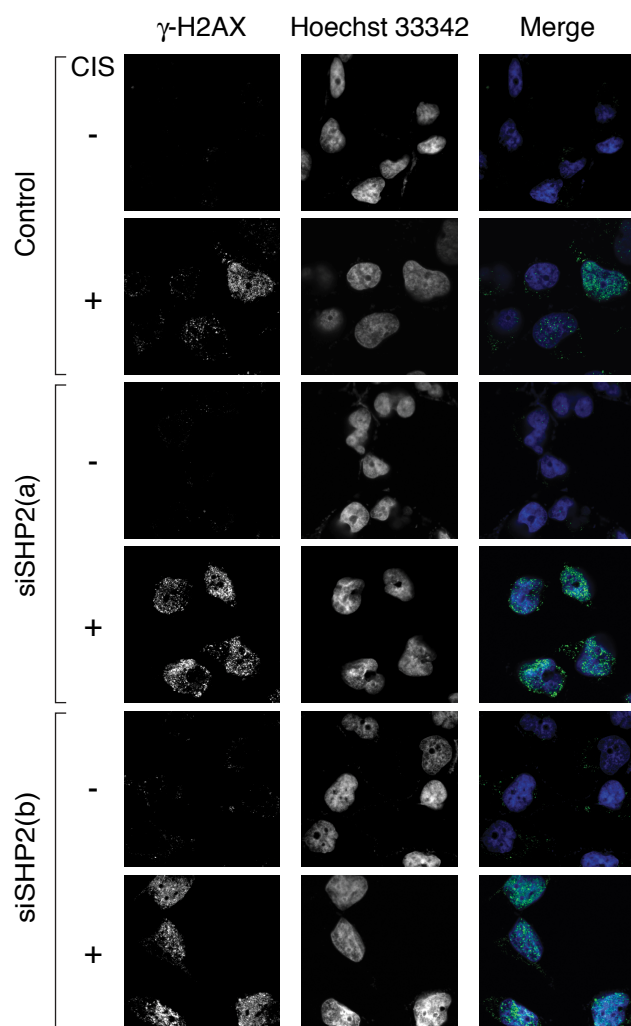

# B

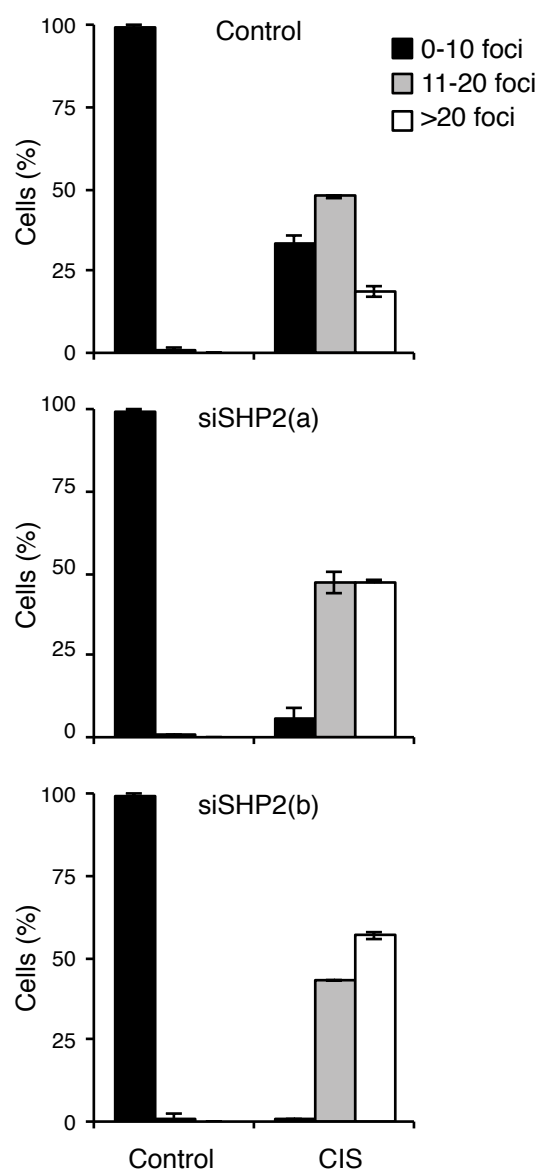

# C

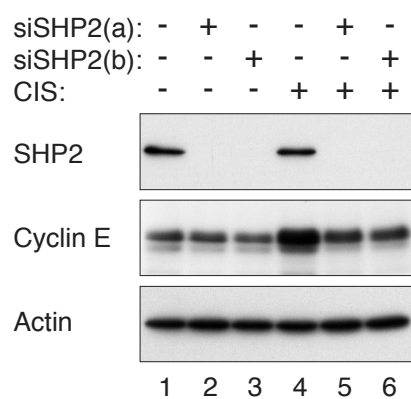

Supplement: Figure S8 — Cells lacking SHP2 are more susceptible to CIS-mediated DNA damage. (A) DNA repair is less effective in SHP2-depleted cells. HeLa cells transfected with control or SHP2 siRNA were treated with CIS. A caspase inhibitor was included to prevent cell death. After 24 h, the cells were fixed and stained for γ-H2AX. Representative images of the γ-H2AX staining are shown. (B) The number of γ-H2AX foci in cells treated in panel A was quantified (n = 50). Mean±SD of three independent experiments is shown. (C) Cells were treated as in panel A. Lysates were prepared and the knockdown of SHP2 was confirmed by immunoblotting. (PDF) [file pone.0049943.s008.pdf]
