# Supplementary material for: Novel Functions of the Phosphatase SHP2 in the DNA Replication and Damage Checkpoints
Source: PLoS One. 2012 Nov 26;7(11):e49943. doi: 10.1371/journal.pone.0049943 (PMC3506573; doi:10.1371/journal.pone.0049943)
Supplement: Table S1 — List of clones in the phosphatome library. The gene symbol, name, and accession number of the genes represented in the library are indicated. The sequences of the two shRNAs for each gene and the z-scores of the hydroxyurea screen are also shown. (PDF) [file pone.0049943.s009.pdf]

Symbol	Full Name	Accession #	Z-score	shRNA (1)	shRNA(2)
ACP1	Acid phosphatase 1, soluble	NM_007099	-0.83	GTCCGTGCTGTTTGTGTGT	GATCCCTATTATGGGAATG
ACP2	Acid phosphatase 2, lysosomal	NM_001610	-0.22	GGACCCCTATCAGGAAGAA	AGGACTTCAGCTTCCGCTT
ACP5	Acid phosphatase 5, tartrate resistant	NM_001611	-1.41	GTGCCCTGGTACGTGCTAG	GGTCCCCAACGGCTATCTG
ACP6	Lysophosphatidic acid phosphatase	NM_016361	-1.73	GGTGGGCATGCAGCAAATG	TGGCCACCGTTTGCTGTTG
ACPL2	Acid phosphatase-like 2	NM_152282	0.68	AGCCCCTTGAACTCCTTGC	GTGGGCTGGCCTTGCTTTA
ACPP	Acid phosphatase, prostate	NM_001099	-0.37	GGGGGTGTCCTGGTCAATG	GAGGCTGCACCCTTATAAG
ACPT	Acid phosphatase, testicular	NM_033068	-0.46	GCCCACGGTCTTCCACTAC	GGACCCCTGGTGTTCGTGG
ALPI	Alkaline phosphatase, intestinal	NM_001631	-0.35	CCCCCGCGGCTTCTACCTC	GGGGCAGAAGAATGGCAAA
ALPL	Alkaline phosphatase, liver/bone/kidney	NM_000478	1.05	GGGTCAGCTCCACCACAAC	CCCCAAAGGCTTCTTCTTG
ALPP	Alkaline phosphatase, placental (Regan isozyme)	NM_001632	0.11	GGGCAGAAGAAGGACAAAC	CCACCACACGAGTGCAGCA
ALPPL2	Alkaline phosphatase, placental-like 2	NM_031313	-4.05	GGGCAGAAGAAGGACAAAC	GGGCAACTTCCAGACCATT
BPGM	2,3-bisphosphoglycerate mutase	NM_001724	-2.58	CAGGGAGCAGATGGCTTTG	TGGGTGCCTGTGGAAAGCT
BPNT1	3'(2'), 5'-bisphosphate nucleotidase 1	NM_006085	0.57	GTGGATCAAGAGCTGATTG	GCCGAGTTCCAGAATCTAT
CDC14A	CDC14 cell division cycle 14 homolog A	NM_033312	-4.72	CCCCCCCTATCTTCCATTC	TCGGGGCTTGTGAGTTCAT
CDC14B	CDC14 cell division cycle 14 homolog B	NM_033332	-11.21	CCCCAGAAGAAGCATATAG	TGGGGTCGAGAATCAAGAT
CDC14C	CDC14 cell division cycle 14 homolog C	BC068452	-0.44	TGGGGTCGAGAATCAAGAC	CCCCAGAAGCAGCATATAG
CDC25A	Cell division cycle 25A	NM_001789	-1.18	GGGGGGATACAAGGAGTTC	GGCCCATGAGACTCTTCAT
CDC25B	Cell division cycle 25B	NM_212530	-3.30	GGCGGCTACAAGGAGTTCT	GGACCTCGTCATGTACAGC
CDC25C	Cell division cycle 25C	NM_022809	-2.01	GGCGGCTACAGAGACTTCT	GACCAGGCAGAAGAGATTTC
CDKN3	Cyclin-dependent kinase inhibitor 3 (CDK2-associated dual specificity phosphatase)	NM_005192	-3.14	GCCGCCCAGTTCAATACAA	GAGCCTATTGAAGATGAAC
CILP	Cartilage intermediate layer protein, nucleotide pyrophosphohydrolase	NM_003613	-1.74	TGGGGAGCCCTACATAGGA	GCCCAGGCCAGACAAGTAT
CTDP1	CTD (carboxy-terminal domain, RNA polymerase II, polypeptide A) phosphatase, subunit 1	NM_004715	-0.33	GGCCTGTGTGCTGAATGTG	ACCGGAAGCTGGTGCTCAT
CTDSP1	CTD (carboxy-terminal domain, RNA polymerase II, polypeptide A) small phosphatase 1	NM_021198	2.31	GGCCCAGGACTCAGACAAG	TGGGCGAGCTCTTTGAATG
CTDSP2	CTD (carboxy-terminal domain, RNA polymerase II, polypeptide A) small phosphatase 2	NM_005730	-1.51	GACCCTCATCCTGGACAAC	GAGGCCTTATGTGGATGAG
CTDSPL	CTD (carboxy-terminal domain, RNA polymerase II, polypeptide A) small phosphatase-like	NM_005808	-1.88	GGACCTGAGTCGCCTTGGG	TTCCCCTGCCTCATACATC
DAPP1	Dual adaptor of phosphotyrosine and 3-phosphoinositides	NM_014395	-1.78	GACCGGAGTAGAAGCTGAT	AGACCAGATGTCACCAGAA
DOLPP1	dolichyl pyrophosphate phosphatase 1	NM_020438	-3.14	CCCGGGCAGAAGCCAGGAA	GCGGGAGCTGCACACGATC
DUPD1	Dual specificity phosphatase and pro isomerase domain containing 1	NM_001003892	-0.58	GGGGAGGAGGAGGACTACT	GCGACGACCACAGTAAGAT
DUSP1	Dual specificity phosphatase 1	NM_004417	-1.28	CGAGGCCATTGACTTCATA	GGACATGCTGGATGCCTTG
DUSP10	Dual specificity phosphatase 10	NM_144728	0.20	GGGGCTTCTCATCCACTGC	CCTGCGGCAGTACTTTGAA
DUSP11	Dual specificity phosphatase 11 (RNA/RNP complex 1-interacting)	NM_003584	2.33	GGCGTGAGGCCAGATGATG	GACTCAGCACATCTCATGC
DUSP12	Dual specificity phosphatase 12	NM_007240	-4.65	ACCCGAGACGGACCTACTC	GTGGACCTATAGCCTTTGC
DUSP13	Dual specificity phosphatase 13	NM_016364	0.32	GGTCCTGGTGCACTGTGTG	TCACCCACGTTGTGAATGC
DUSP14	Dual specificity phosphatase 14	NM_007026	-0.22	GTGCCTCTGGCTGACATGC	GCCCGGCGACCTGTCATCA
DUSP15	Dual specificity phosphatase 15	NM_177991	-1.93	CCCCAACCCAGGCTTTAGG	TGGGGGGAACTGCCTTGTG
DUSP16	Dual specificity phosphatase 16	NM_030640	-0.58	GTGGGCAGTCAGTCTAGCT	GGGCTGGCACTCGGATATC
DUSP18	Dual specificity phosphatase 18	NM_152511	-0.07	GCAGGGCCGTACTTTGCTG	TGGTGTGGCCGCCAACAAC
DUSP19	Dual specificity phosphatase 19	NM_080876	-2.64	TGCACCAGGGTGACAACGC	GAAGTAGAGCCGAGCAGTG
DUSP2	Dual specificity phosphatase 2	NM_004418	0.82	CCAGATGGTGGAGATCAGT	CGCACGCTGCTGCTGGACT
DUSP21	Dual specificity phosphatase 21	NM_022076	-0.55	GGACCTACGTACGATGATA	CACCGTGCGCATGATCAAC
DUSP22	Dual specificity phosphatase 22	NM_020185	-2.94	CCCCAACGTGGGCTTCCAG	GATCCTGCCCGGCCTGTAC
DUSP23	Dual specificity phosphatase 23	NM_017823	-2.60	AGCAGTCTTCCAGTTCTAC	CTTCTCCTGGGTGCTTCCG
DUSP24	Serine/threonine/tyrosine interacting-like 1	NM_016086.2	-1.28	GGGGGCTATGAGCGCTTCT	TGGGAGAAGACTATCCTTG
DUSP26	Dual specificity phosphatase 26	NM_024025	-1.06	AGACCACCGAGGCATCATC	GGTCTCCTGTTCGAACTCG
DUSP3	Dual specificity phosphatase 3 (vaccinia virus phosphatase VH1-related)	NM_004090	-6.17	AGGGCTGCCGACTTCATTG	GGACTCCGGCATCACATAC
DUSP4	Dual specificity phosphatase 4	NM_001394	-0.89	GGACACTATCAGTACAAGT	ACGGGTGAGGCTGGAGGAG
DUSP5	Dual specificity phosphatase 5	NM_004419	4.29	GGTCCTGGTCCACTGTGAG	GGGGAGGCAGCAGGCTCTT
DUSP6	Dual specificity phosphatase 6	NM_022652	-1.16	GACGGTGGCGTGGCTCAAC	CAGGGTTCCAGCACAGCAG
DUSP7	Dual specificity phosphatase 7	NM_001947	-1.91	CCTGGACGTGCTCGGCAAG	GCCCGCTCCAAGAAGTGTG
DUSP8	Dual specificity phosphatase 8	NM_004420	-1.52	CCGCCTCAAGCGCTCCTTC	GCGCCGCAGCTGCCAGATG
DUSP9	Dual specificity phosphatase 9	NM_001395	-2.40	CCTCCCAAACTTCTTCGAG	GCTCCACCTCTCTCTCAAC
DUT	DUTP pyrophosphatase	NM_001948	-2.62	ACGGACATTCAGATAGCGC	GGTGATCGAATTGCACAGC
ENPP1	Ectonucleotide pyrophosphatase/phosphodiesterase 1	NM_006208	-2.39	GGGCGACTGCTGCATCAAC	GCCCTCTTTGTTGGCTATG
ENPP2	Ectonucleotide pyrophosphatase/phosphodiesterase 2 (autotaxin)	NM_006209	-2.10	GGGGTGAAAGCTGGAACAT	CGAGGAGAGCTGCAATAGC
ENPP3	Ectonucleotide pyrophosphatase/phosphodiesterase 3	NM_005021	-2.70	GGCCTGAAGCAGCGGAATT	CGTGGAGAGCTGTCCTGAA
EPM2A	Epilepsy, progressive myoclonus type 2A, Lafora disease (laforin)	NM_001018041	-0.58	GAGGCCGGCTGTCTACATT	TCTGAGGAAGGTGCAGTAT
EYA1D	Eyes absent homolog 1, transcript variant 4	NM_172059	-0.93	GAGGGAAGCCTGGCTGCAG	CCTGGACAGGCACCATACA
EYA2V	Eyes absent homolog 2, transcript variant 5	NM_172110	-2.47	GGGAGACCTGGCTACAGCT	GAGGGAGACACAGACAGGC
EYA3B	Eyes absent homolog 3, transcript variant 2	NM_172098	-2.97	CCGGGGCAAGAGGAAAGCT	GCCAAGATGCAGGAATCAG
EYA4B	Eyes absent homolog 4, transcript variant 2	NM_172103	-0.57	GAGGGATGCCTGGCTACAG	TGGGACAGGAGTGTCTCTT
FBP1	Fructose-1,6-bisphosphatase 1	NM_000507	0.04	CCCCATGGCCTACGTCATG	GGAGGCCGTGTTAGACGTCAT
FBP2	Fructose-1,6-bisphosphatase 2	NM_003837	-2.28	AGGGGCGTCAGGCCAAAGG	GCCCCTTCGAAACCGACAT
FLJ30092	AF-1 specific protein phosphatase	BC021144	-1.52	CCTCCTCCACCTCTTCTTC	GCCCATCAGAGTCTCTGAC
G6PC	Glucose-6-phosphatase, catalytic (glycogen storage disease type I, von Gierke disease)	NM_000151	-4.15	CCCCCATCCCAAGTCGAGC	AGCCGACCTACAGATTTCG
G6PC2	Glucose-6-phosphatase, catalytic, 2	NM_021176	0.48	CGGCCAGTCTGGGCACATA	CCCCGACTGGATCCACATT
G6PC3	Glucose 6 phosphatase, catalytic, 3	NM_138387	-0.74	GCACCGCCCATCCACTCTT	GTGGTGTGAGCGGCCTGAG
ILKAP	integrin-linked kinase-associated serine/threonine phosphatase 2C	NM_030768	-2.76	CCTCCGAGGAAGAGAAGAA	GGACCCCTGCTCTTTGATG
IMPA1	inositol(myo)-1(or 4)-monophosphatase 1	NM_005536	-1.03	CCCCACATGGATCATTGAC	CTGGCGGAGCAGATGCATA
IMPA2	Inositol(myo)-1(or 4)-monophosphatase 2	NM_014214	-1.98	GGCCTTGGTTCTGACAGAA	GCTCCACATTGGCACTCTG
IMPAD1	Inositol monophosphatase domain containing 1	NM_017813	-1.86	AGAGGTACCAGCAGAAAGT	GCCCGCTCTTCCTACAATG
INPP1	Inositol polyphosphate-1-phosphatase	NM_002194	-3.77	GGGAGGACTCATTGCATAC	TGGGACTCTTGTGCTGCTC
INPP4A	Inositol polyphosphate-4-phosphatase, type I, 107kDa	NM_004027	-1.04	TGGGGTCCGGTTCACCAGC	GGAGATCATCGCCCAGATC
INPP4B	Inositol polyphosphate-4-phosphatase, type II, 105kDa	NM_003866	-1.47	GTGGGAGAGCTGCTGAAGT	GGCCAGGAGGCATTCTTAA
INPP5A	Inositol polyphosphate-5-phosphatase, 40kDa	NM_005539	-0.70	GGTCGCTGGCAAAGAGATC	GCCCGTGTTCCTGGCCTTC
INPP5B	Inositol polyphosphate-5-phosphatase, 75kDa	BC058932	-0.91	CACCAGCATCTGCGTTGTG	TGGGAAGGGAGTGCCTATG
INPP5D	Inositol polyphosphate-5-phosphatase, 145kDa	NM_005541	-2.47	GGCGGAGGAGCTGCTTTCC	GCCCGAGATGTTTGAGAAC
INPP5E	Inositol polyphosphate-5-phosphatase, 72 kDa	NM_019892	-2.96	CCCCTATCGCTCCAGCGCA	GGAGATTCAGAGGCAGCAA
INPP5F	Inositol polyphosphate-5-phosphatase F	NM_198331	-0.28	ACTCCCAGGAGACCATGAG	GGACCACTACATCCTGCAG
INPPL1	Inositol polyphosphate phosphatase-like 1	NM_001567	-1.48	GGACCGGACTCAGCGCAAG	GTCCCAGCGTGTCCAGAAC
ITPA	Inosine triphosphatase (nucleoside triphosphate pyrophosphatase)	NM_181493	-2.36	GGCGGAGAAGAACGCTGTC	GTCAGCCTATGCGCTCTGC
LHPP	Phospholysine phosphohistidine inorganic pyrophosphate phosphatase	NM_022126	-2.31	GCCCACCAGGCCGTCATGA	GAGGGCGTTACTACAAGGA
LPPR2	Lipid phosphate phosphatase-related protein type 2	NM_022737	-1.67	GCCCCCACCATGGATAGCC	GGGCTCCCGCCTGGTCAAA
MINPP1	Multiple inositol polyphosphate histidine phosphatase, 1	NM_004897	-0.44	AGGGACGGCAGGATATGCG	GCACCGCTGCATGGATAGC
M-RIP	Myosin phosphatase-Rho interacting protein	NM_201274	0.42	GCGGGACTTCACCAATGAA	GGAGGCCTCAGACCTTCTG
MTM1	Myotubular myopathy 1	NM_000252	1.87	CCCCAGGATCAAGCAACAA	TGGGAGGCGCGACAAGTAG
MTMR1	myotubularin related protein 1	NM_176789	-4.43	CGGACAGGCACTATTTGCA	GGGTGGAGGATATGAAAGT
MTMR10	Myotubularin related protein 10	NM_017762.2	-3.00	ACCGACAGCCAGAACAGTG	CGACCACAAGAGGAAGCAG
MTMR11	Myotubularin related protein 11	NM_181873.2	-1.72	TGGACACCAAGAATATCTC	GGCCAGTGACATTTCAGTA
MTMR12	Myotubularin related protein 12	NM_019061	-1.41	GGCCAGGATACACAAAGCA	TACCCTGAGAAGCTCATCA
MTMR12	Myotubularin related protein 12	NM_001040446	0.43	GGCCAGGATACACAAAGCA	TACCCTGAGAAGCTCATCA
MTMR13	Myotubularin related protein 13 / SET binding factor 2	NM_030962.3	0.74	CCCAGTGAAGATGATAAAG	GCCCGGGTTCAGGAATTAA
MTMR14	Myotubularin related protein 14	NM_001077526	-0.48	GACCCAGAGGAGGAAGAGT	GGGCAAGCACATTTGCAGG
MTMR15	Myotubularin related protein 15	NM_001146094	-1.69	CCGGTCATCCTTACTACCT	CCGGCACCTTGATGAAATG
MTMR2	Myotubularin related protein 2	NM_201281	-2.04	GGTGGAATCCACGGATGAA	ACCCACTGGTTGTCTAACT
MTMR3	Myotubularin related protein 3	NM_153051	-2.75	GAGGGGCTTGTGTGCAATG	TGGGGGAGACCTTTCTGAC
MTMR4	Myotubularin related protein 4	NM_004687	-1.06	GGGTGGAGGCTGTGAATGT	AGCCCGTTCCACTGAACTG
MTMR6	Myotubularin related protein 6	NM_004685	-1.51	CCGGGACTACAAGATTTGT	TGGCACTAAAGGGCTTTCT
MTMR7	myotubularin related protein 7	NM_004686	-2.62	TCGGGCCGACTACCTGAAT	GGGGCAAGTGTGCTTGTTC
MTMR8	Myotubularin related protein 8	NM_017677	-0.99	CCCAGGGAGCCAGTTTATG	GGAGGCAGACTACTCCAAG
MTMR9	Myotubularin related protein 9	NM_015458	-3.85	TCCCCTCTTTGAAGCCAAC	GGAGGTGGCTTTGAACAAG
OCRL	Oculocerebrorenal syndrome of Lowe	NM_000276	-3.56	CTGGACTTGAGCACAGAAG	TCCCTGATGAGGAACACTG
PDP2	Pyruvate dehydrogenase phosphatase isoenzyme 2	NM_020786	-0.93	GGGGGTAGACGCTTATACT	GAGGGAGCACCCTGAGTCA
PDPR	Pyruvate dehydrogenase phosphatase regulatory subunit	NM_017990	0.34	CCGGGGAGAGTATGAGATT	TGGGGTGGAAGGATATTGT
PDXP	Pyridoxal (pyridoxine, vitamin B6) phosphatase	NM_020315	-0.23	GGCGGCTCTGTTTGTGAGC	GCCCAGCCCCTACATGTTC
PFKFB1	6-phosphofructo-2-kinase/fructose-2,6-biphosphatase 1	NM_002625	-1.65	CCAGGTTGCAGAAGATCTG	GGCCCTGAATGAGATTGAT
PFKFB2	6-phosphofructo-2-kinase/fructose-2,6-biphosphatase 2	NM_001018053	-4.11	CTCCCCGACTCTGATCGTT	GCCCTGACTATCCTGAAAG
PFKFB3	6-phosphofructo-2-kinase/fructose-2,6-biphosphatase 3	NM_004566	0.88	GAGGATCAGTTGCTATGAA	GCCTCGCATCAACAGCTTT
PFKFB4	6-phosphofructo-2-kinase/fructose-2,6-biphosphatase 4	NM_004567	-2.48	GGTCCTCAACGAGATCGAT	CCCCCTGAAGAAGATCTGG
PHLPP	PH domain and leucine rich repeat protein phosphatase	NM_194449	-5.24	GCCCTGTATGGTGTGTTTG	TCCCTTCCTGCTGAGTTGG
PHLPPL	PH domain and leucine rich repeat protein phosphatase-like	NM_015020	-2.43	CGGCCTGGACAGTGATGAT	CGACCTCTTCAGATCGTTT
PHOSPHO1	Phosphatase, orphan 1	NM_178500	-0.88	GGTGCTCAGCGACTACCTG	CGAGTACATGCAGCGCGTC

PHPT1	Phosphohistidine phosphatase 1	NM_014172	-1.95	GGAGATCGTGCGCGGCTAC	GTGGGCTGAGTACCATGCG
PIB5PA	Phosphatidylinositol (4,5) bisphosphate 5-phosphatase, A	NM_014422	-0.89	GGCGGAGCAGCGCAAAGAC	GGGCCATGGAGACTTCATC
PME-1	Protein phosphatase methylesterase 1	NM_016147	-3.08	CCTGGTACCAAGCCTCTTG	TGTGGTTGAAGCCATGTAT
PNKP	Polynucleotide kinase 3'-phosphatase	NM_007254	-1.93	ACCCCGGCTGGGAGAACTT	CGGGTCGCCATCGACAACA
PPAP2A	Phosphatidic acid phosphatase type 2A	NM_003711	-0.74	ACACCACTGGAGCGATGTG	GGGAGACTGGGCAAGACTC
PPAP2B	Phosphatidic acid phosphatase type 2B	NM_003713	-4.79	CCCCTACGTGGCAGCACTC	GTGGGCTGCTTCCTCTTTG
PPAP2C	Phosphatidic acid phosphatase type 2C	NM_177526	-0.53	GGAGGAGGAGCTGGAACGG	CTACGTGGCTGCTGTATAC
PPEF1	Protein phosphatase, EF hand calcium-binding domain 1	NM_006240	-6.44	CCAGGAGATCTGACACATC	GCCCGAAGGGTATGAAATC
PPEF2	Protein phosphatase, EF hand calcium-binding domain 2	NM_006239	-3.32	GAGGGCTGCAAGGCCAACA	CCGGGTCTCAACCTGTTAC
PPFIA1	PTPRF interacting protein (liprin), alpha 1	NM_177423	1.99	GGGACCGCCTTCTTGATAC	CCCCAGCAGTAGCAACAGT
PPFIA2	PTPRF interacting protein (liprin), alpha 2	NM_003625	-2.42	CACCCAGGCAAGGCAGATT	GCCCCCAAGAAGAAAGGAA
PPFIA3	PTPRF interacting protein (liprin), alpha 3	NM_003660	-1.71	GCCCCCAAGAAGAAGAGCA	CTCCCGACTCAGCTGAGAT
PPFIA4	PTPRF interacting protein (liprin), alpha 4	NM_015053	4.73	CCCCAGCAGCAGCAACAGC	CACCCAGGCACGCCAAGTG
PPM1A	Protein phosphatase 1A (formerly 2C), magnesium-dependent, alpha isoform	NM_177952	1.14	GGAGGCAGAGTTGGACAAG	GGTCCTACTGAGCAGCTTG
PPM1B	Protein phosphatase 1B (formerly 2C), magnesium-dependent, beta isoform	NM_002706	-2.67	AGCCCTGCTGAACCAGCTG	GGGCCCAACAGAACAACTT
PPM1D	Protein phosphatase 1D magnesium-dependent, delta isoform	NM_003620	-7.24	CCTCACAGCGAAAGAACTC	CGACCTCGACTCACTCACAA
PPM1E	Protein phosphatase 1E (PP2C domain containing)	NM_014906	-2.38	GACCCAGGCTACCTAGATC	GGGCCAAGCTGTTGAACTA
PPM1F	Protein phosphatase 1F (PP2C domain containing)	NM_014634	-0.84	GGCCCCTGTGACCTTGCTG	GGCGCGCATTGAAGCATTG
PPM1G	Protein phosphatase 1G (formerly 2C), magnesium-dependent, gamma isoform	NM_177983	6.41	TGGGGAGCTTCGGTTATTG	GCCCCGAAACACAGCAGAG
PPM1K	Protein phosphatase 1K (PP2C domain containing)	NM_152542	-3.36	GGAGAGTGCTGCTAAGCTC	GACCAGTGGTGTCATAGCA
PPM1L	Protein phosphatase 1 (formerly 2C)-like	NM_139245	-2.25	GCAGGCACAACGTGTTTGA	GACCTGGGAGTTCAAGAAC
PPM1M	Protein phosphatase 1M (PP2C domain containing)	NM_144641	-1.59	GGGGGATGACTTGGGACAG	GCCCTTCTTGCTCTCTGTG
PPM2C	Protein phosphatase 2C, magnesium-dependent, catalytic subunit	NM_018444	-3.17	GGCCACAGGATAAGTTTCT	GGAGGCATCCAAATTGTAC
PPP1CA	Protein phosphatase 1, catalytic subunit, alpha isoform	NM_206873	-2.71	GACGGCTACGAGTTCTTTG	GCCCGCCGACAAGAACAAG
PPP1CB	Protein phosphatase 1, catalytic subunit, beta isoform	NM_002709	-2.16	GAGGAAACCATGAGTGTGCTA	GTCTCGGGAGATCTTTCTC
PPP1CC	Protein phosphatase 1, catalytic subunit, gamma isoform	NM_002710	-1.70	GGGGTATGATCACAAAGCA	TGTCCAGCTTCAGGAGAAT
PPP2CA	Protein phosphatase 2 (formerly 2A), catalytic subunit, alpha isoform	NM_002715	0.61	TGGCCTCACGTTGGTGTCTAG	GAGCCTCTGCGAGAAGGCTAA
PPP2CB	Protein phosphatase 2 (formerly 2A), catalytic subunit, beta isoform	NM_004156	-2.94	GAGCCCTGGATCGTTTACA	TGGTCTCACACTGGTTTCT
PPP3CA	Protein phosphatase 3 (formerly 2B), catalytic subunit, alpha isoform (calcineurin A alpha)	NM_000944	-1.92	ACCCTGCAAAGCGCTACTG	GGAGGGAAGGCTGGAAGAG
PPP3CB	Protein phosphatase 3 (formerly 2B), catalytic subunit, beta isoform (calcineurin A beta)	NM_021132	-0.51	GTGCCACAGTTGAGGCTAT	GACCAGTTTGATGGTTCAG
PPP3CC	Protein phosphatase 3 (formerly 2B), catalytic subunit, gamma isoform (calcineurin A gamma)	NM_005605	-0.23	CCTCAGCACACTCACATGC	GCCCAAGATGCTGGGTATC
PPP4C	Protein phosphatase 4 (formerly X), catalytic subunit	NM_002720	-1.92	GCCCGTGGCCGACTACTTC	GGCCAGAGAGATCTTGGTA
PPP5C	Protein phosphatase 5, catalytic subunit	NM_006247	-3.85	CGGGCGCTCGATCAGCAAG	GCCCCATGACAAGGATGCC
PPP6C	Protein phosphatase 6, catalytic subunit	NM_002721	0.09	CGACCTGAAGCGGCTATGT	TGCCTGGAGATACTGTACC
PPTC7	PTC7 protein phosphatase homolog	NM_139283	-0.69	GGACGGTTCGTACCTAGTA	TGCGGACGTGTGAACGTTT
PSPH	Phosphoserine phosphatase	NM_004577	-1.31	GTCGCCTACAGGAGCGAAA	GCCTGTCCTCCTGCTGATG
PSTPIP1	Proline-serine-threonine phosphatase interacting protein 1	NM_003978	-0.28	GTCCCAAGACCACTTCGTT	GGCCATGGAGTCCAAGAAG
PSTPIP2	Proline-serine-threonine phosphatase interacting protein 2	NM_024430	-2.81	TGGCCGCAAGAACTGCAAA	CACCCATCATGTATGAGAA
PTEN	Phosphatase and tensin homolog (mutated in multiple advanced cancers 1)	NM_000314	-1.46	CCCACCACAGCTAGAACTT	GGGACGAACTGGTGTAATG
PTENP1	Phosphatase and tensin homolog (mutated in multiple advanced cancers 1), pseudogene 1	BC038293	-1.48	CCCACCACAGCTAGAACTT	TCCAGAGGCTAGCAGTTCA
PTP4A1	Protein tyrosine phosphatase type IVA, member 1	NM_003463	-1.74	TGCGGCTGCGTTTCAAAGA	CCGCCCAGCTCCTGTGGAAGT
PTP4A2	Protein tyrosine phosphatase type IVA, member 2	NM_080391	0.15	GGGGAGCGTTCAATTCCAA	GAGCCAGGTTGCTGTGTTG
PTP4A3	Protein tyrosine phosphatase type IVA, member 3	NM_007079	-1.10	GACCCACACACGCACAAGA	CCCCACCAACGCCACGCTC
PTPDC1	Protein tyrosine phosphatase domain containing 1	NM_152422	-1.46	GGGGAGACTCCACAGACAG	GGGACAGCACCAGACTATT
PTPLA	Protein tyrosine phosphatase-like (proline instead of catalytic arginine), member a	NM_014241	0.48	GGTGCTTCATGGAGAGGTG	TATACGCTGCCTTGCCGCA
PTPLB	Protein tyrosine phosphatase-like (proline instead of catalytic arginine), member b	NM_198402	0.85	TGGGAGTGTCAGGAGAACT	GGGTAGCTACCATAGCCTTTA
PTPMT1	Protein tyrosine phosphatase, mitochondrial 1	BC018974	-1.85	TCCCTTGCTAGACAGCTTC	GGTGCTGCTTACCAATTTG
PTPN1	Protein tyrosine phosphatase, non-receptor type 1	NM_002827	-1.26	TGGGAAATGCAGGGAGTTC	GACCCTTCTTCCGTTGATA
PTPN11	Protein tyrosine phosphatase, non-receptor type 11 (Noonan syndrome 1)	NM_002834	3.11	GCGCCGCTCATGACTATAC	CTCCCGAGTGATTGTCATG
PTPN12	Protein tyrosine phosphatase, non-receptor type 12	NM_002835	0.07	TGGCCTGCCGAGAATTTGA	GGACCCGCAGTTGCCTTGT
PTPN13	Protein tyrosine phosphatase, non-receptor type 13 (APO-1/CD95 (Fas)-associated phosphatase)	NM_080684	-1.59	GGACCACTGCCTACAACTG	GGTCCGTCGCAGCCATTGA
PTPN14	Protein tyrosine phosphatase, non-receptor type 14	NM_005401	-6.31	GGACCGGCGTGCTCATTCT	GGACGGTGTGGCATTTACA
PTPN18	Protein tyrosine phosphatase, non-receptor type 18	NM_014369	-2.22	GAGGGACACAGCGACTACA	CCCCTCTGTGTCCACTGCA
PTPN2	Protein tyrosine phosphatase, non-receptor type 2	NM_002828	-2.20	GGCCACCACAGCTCAGAAG	GCCCATATGATCACAGTCG
PTPN20A	Protein tyrosine phosphatase, non-receptor type 20A	NM_015605	2.09	GTGGCAGTGATCCCAGCAT	CTCTGGGAATCAACCAAGC
PTPN21	Protein tyrosine phosphatase, non-receptor type 21	NM_007039	-1.41	ACCCCAGCCCTACGTGATG	GCCCCAACCCTCCGTTGTT
PTPN22	Protein tyrosine phosphatase, non-receptor type 22	NM_012411	0.80	CCAGGAGTCAGCTGTACTA	TGGGGTGGAACATCTGAAC
PTPN23	Protein tyrosine phosphatase, non-receptor type 23	NM_015466	-3.05	CCGGCACCAGGATGTCATG	CCCCGACACTGCCTCACTG
PTPN3	Protein tyrosine phosphatase, non-receptor type 3	NM_002829	1.08	GCCCGCACCAAGAGAGTTT	CCGGTGTGTTGGTCACTAT
PTPN4	Protein tyrosine phosphatase, non-receptor type 4	NM_002830	-1.11	GAGGGCTGGCAAGGAAGAA	GGTGGCTGGATCCAAACAA
PTPN5	Protein tyrosine phosphatase, non-receptor type 5	NM_032781	-0.56	CCCTCACAGCAGAGTGTGT	ACCTCGTCTCCTCTTTGCT
PTPN6	Protein tyrosine phosphatase, non-receptor type 6	NM_002831	-1.05	GGGCAAGAACCGCTACAAG	GGGCCTGGACTGTGACATT
PTPN7	Protein tyrosine phosphatase, non-receptor type 7	NM_002832	-1.53	TCCCCAGAGCCGTGTCTGT	ACGCCAGCCAAGAAGCATG
PTPN9	Protein tyrosine phosphatase, non-receptor type 9	NM_002833	-1.43	GGCGGCAGGAGAAAGTGTG	GGCCATCCTGGAGTTCGCAG
PTPNS1	Protein tyrosine phosphatase, non-receptor type substrate 1	NM_080792	-1.24	TGGGAATGAGCTCTCAGAC	GTCCGTGTTGGTTGCAGCT
PTPNS1L2	Protein tyrosine phosphatase, non-receptor type substrate 1-like 2	NM_178460	-2.34	TGGACCTGTCTTGTGGTTC	CAGGGCCAAACCGGAAATT
PTPNS1L3	Protein tyrosine phosphatase, non-receptor type substrate 1-like 3	XM_209363	0.33	CACCTTAGCATGGAGCAAG	TGGGTGAAGGTGAGCACTC
PTPRA	Protein tyrosine phosphatase, receptor type, A	NM_080840	-3.53	CGGCCGCACTGAGGATGTG	GCGGGGCGAAGAGAATACA
PTPRB	Protein tyrosine phosphatase, receptor type, B	NM_002837	-0.91	AGGGACCATCAAGGAATCC	AGGGATCCTAGTTCATGGC
PTPRC	Protein tyrosine phosphatase, receptor type, C	NM_080921	-1.17	GGCGACAGAGATGCCTGAT	GGTCCCAGGGATGAAACTG
PTPRCAP	Protein tyrosine phosphatase, receptor type, C-associated protein	NM_005608	-1.10	TGACCTTGAGCGACAGGAG	GCCAGAGACAGTGACACGG
PTPRD	Protein tyrosine phosphatase, receptor type, D	NM_130393	-2.37	ACCCACGGACTCGTTCAAG	CCTCCAGGAACTCCTGTAG
PTPRE	Protein tyrosine phosphatase, receptor type, E	NM_130435	1.35	GGTCCCAAACAGGAAACGG	TCCCACACTATCGTGATGC
PTPRF	Protein tyrosine phosphatase, receptor type, F	NM_130440	-4.42	TCCGGCAGTTCCAGTTCAC	GGAGGATGAGATGCCAGTT
PTPRG	Protein tyrosine phosphatase, receptor type, G	NM_002841	-2.01	GGGTGTCGTACATCATGAG	GGGTCGTCAGAATGAAAGG
PTPRH	Protein tyrosine phosphatase, receptor type, H	NM_002842	-0.07	CCCGGGTGTTTGTATGCGT	CCCGGGACGTTGTACAATT
PTPRJ	Protein tyrosine phosphatase, receptor type, J	NM_002843	0.18	GGGGCATCTCGGACAGTTT	GGGACGTTCTCAGAGCTTG
PTPRK	Protein tyrosine phosphatase, receptor type, K	NM_002844	-1.81	GGACCTCTTGCCAATCCAA	CCCAGTGCGTACGCATTGC
PTPRM	Protein tyrosine phosphatase, receptor type, M	NM_002845	-4.00	GGAGGAATACGAGAGCTTC	GGGGTCGTAGACATCTACA
PTPRN	Protein tyrosine phosphatase, receptor type, N	NM_002846	-0.40	GGGACAGGTCTGGCTTGGC	GCCCCTGCCCTCTTCAGCA
PTPRN2	Protein tyrosine phosphatase, receptor type, N polypeptide 2	NM_130843	-2.35	GGAGGAGCAGAGCCTTCCA	GGCCCCTCGACATGGAGAG
PTPRO	Protein tyrosine phosphatase, receptor type, O	NM_030670	-2.19	CCCGGTTCAACTGGATGAC	GAGGAAGGTGCAGACTACA
PTPRQ	Protein tyrosine phosphatase, receptor type, Q	XM_926134	-0.30	GGTCCACTACCAGGAACAG	GGACCCACCTAACAACATG
PTPRR	Protein tyrosine phosphatase, receptor type, R	NM_130846	0.58	TCCCCTCAGCAGAGTGTGT	CCAGGAGATCCACCTATCG
PTPRS	Protein tyrosine phosphatase, receptor type, S	NM_130853	-0.42	GCCCCACACCTTCTACAAC	GGCCCACACGTCACGCTTC
PTPRT	Protein tyrosine phosphatase, receptor type, T	NM_007050	-2.48	GGAGGAGCGACTTCAGAAG	GGGGTGGTGGACATCTTCA
PTPRU	Protein tyrosine phosphatase, receptor type, U	NM_133178	-0.11	GGGGGAGACCCGGCTGAAT	GGGCCGAAGCCTGAGATGG
PTPRZ1	Protein tyrosine phosphatase, receptor-type, Z polypeptide 1	NM_002851	-2.15	GGCCCACTGAAATCCACAG	TGCCCAACTGGTGGTTATG
RNGTT	RNA guanylyltransferase and 5'-phosphatase	NM_003800	-2.13	ACCCTGTCACCAAGGAGAT	TGGGAGGAGAAGGGTTACT
SGPP1	Sphingosine-1-phosphate phosphatase 1	NM_030791	-1.98	GTGGTGCTGGAATTGCATG	CTGGCCGCTCTACTGCCTG
SIRPA	Signal-regulatory protein alpha	NM_080792	-3.46	TGGGAATGAGCTCTCAGAC	GTCCGTGTTGGTTGCAGCT
SIRPD / SIRPB1	Signal-regulatory protein beta 1	NM_006065	-1.35	GTCCGTATCAGTTGCAGCT	TGGGAATGAGCTCTCAGAC
SKIP	Skeletal muscle and kidney enriched inositol phosphatase	NM_130766	-0.42	GGTCTCCTGCAGCGACAAC	GGACCAGCTCAGCATTGCC
SNAP23	Synaptosomal-associated protein, 23kDa	NM_003825	-2.96	TGCCAGAGCAAAGAAACTC	TCCTGGGTTTAGCCATTGA
SPAP1 / FCRL2	Fc receptor-like 2	NM_030764	-0.12	TGTGGGCTCTGTAGATGTG	CCTCTCTTTGACTGCAGAA
SSH1	Slingshot homolog 1	NM_018984.2	-0.62	GGACCTGAGGCTTCTGTTC	CTCGGAGAACCTAAACAAC
SSH2	Slingshot homolog 2	NM_033389.2	-0.14	GTCGCCAGAGCGCATAACT	GGCCTGTGTACCAAAGAAA
SSH3	Slingshot homolog 3	NM_017857.2	0.11	CAGGGTCACCCACATCTTG	GGTCTGAGCCAGGATGAG
SYNJ1	Synaptojanin 1	NM_203446	-2.23	TGGGAGGTGCAACTGGAAA	TCCGAGGATCTGTTCCATT
SYNJ2	Synaptojanin 2	NM_003898	-1.60	GGTGGGAAGCTAGAGAAAT	GGGCAGATGCTGGTAACTT
TA-PP2C	T-cell activation protein phosphatase 2C	NM_139283	-0.74	GTCCCTTTGCTCGGTAGCA	GGACGGTTCGTACCTAGTA
TENC1	Tensin like C1 domain containing phosphatase	NM_198316	-2.07	CCCCAAGGTTCAAGACTTC	ACCCCAGAGCCTCCTACAC
TPTE	Transmembrane phosphatase with tensin homology	NM_199260	-1.52	GGAGGCACAGATAGAACAG	TGTCCCCACTCTACATCAG
TPTE2	Transmembrane phosphoinositide 3-phosphatase and tensin homolog 2	NM_199255	-1.46	CCGGGACTATGGTTTGTGC	TGTCCCCACTCTACATGAG
